# Combinatorial synthesis enables scalable designer detergents for membrane protein studies†

**DOI:** 10.1039/d2sc03130b

**Published:** 2022-08-30

**Authors:** Leonhard H. Urner, Armin Ariamajd, Alex Weikum

**Affiliations:** TU Dortmund University, Department of Chemistry and Chemical Biology Otto-Hahn-Straße 6 44227 Dortmund Germany leonhard.urner@tu-dortmund.de; Freie Universität Berlin, Institute of Chemistry and Biochemistry Takustraße 3 14195 Berlin Germany

## Abstract

Non-ionic detergents with tailor-made properties are indispensable tools for today's world applications, such as cleaning, disinfection, and drug discovery. To facilitate their challenging production, herein we introduce a new detergent class, namely scalable hybrid detergents. We report a combinatorial synthesis strategy that allows us to fuse head groups of different detergents into hybrid detergents with unbeatable ease. Importantly, combinatorial synthesis also enables the choice between (i) high-throughput preparation of detergents for small scale applications and (ii) large scale preparation of individual detergents. This combinatorial synthesis strategy enables an unprecedented fine tuning of detergent properties, such as overall polarity and shape, which are determining factors in applications, such as membrane protein research. Our data show that membrane protein purification parameters, such as protein yields and activity, can be linked to overall polarity and shape. Conveniently, both parameters can be theoretically described by means of the hydrophilic–lipophilic balance (HLB) and packing parameter concepts. Both concepts are principally applicable to all non-ionic detergent classes, which facilitates the identification of widely applicable design guidelines for the predictable optimization of non-ionic detergents. Our findings permit access to a yet unexplored chemical space of the detergentome, therefore creating new possibilities for structure–property relationship studies. Seen from a broader perspective, combinatorial synthesis will facilitate the preparation of designer detergents with tailor-made properties for future applications in today's world.

## Abstract 2

Combinatorial detergent synthesis permits access to an unexplored part of the detergentome and provides new directions for the preparation of custom-made detergents for future applications.

## Graphical abstract

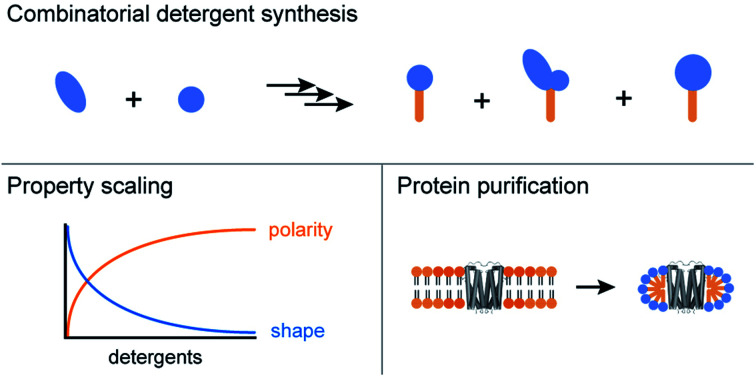

## Introduction

**Fig. 1:**
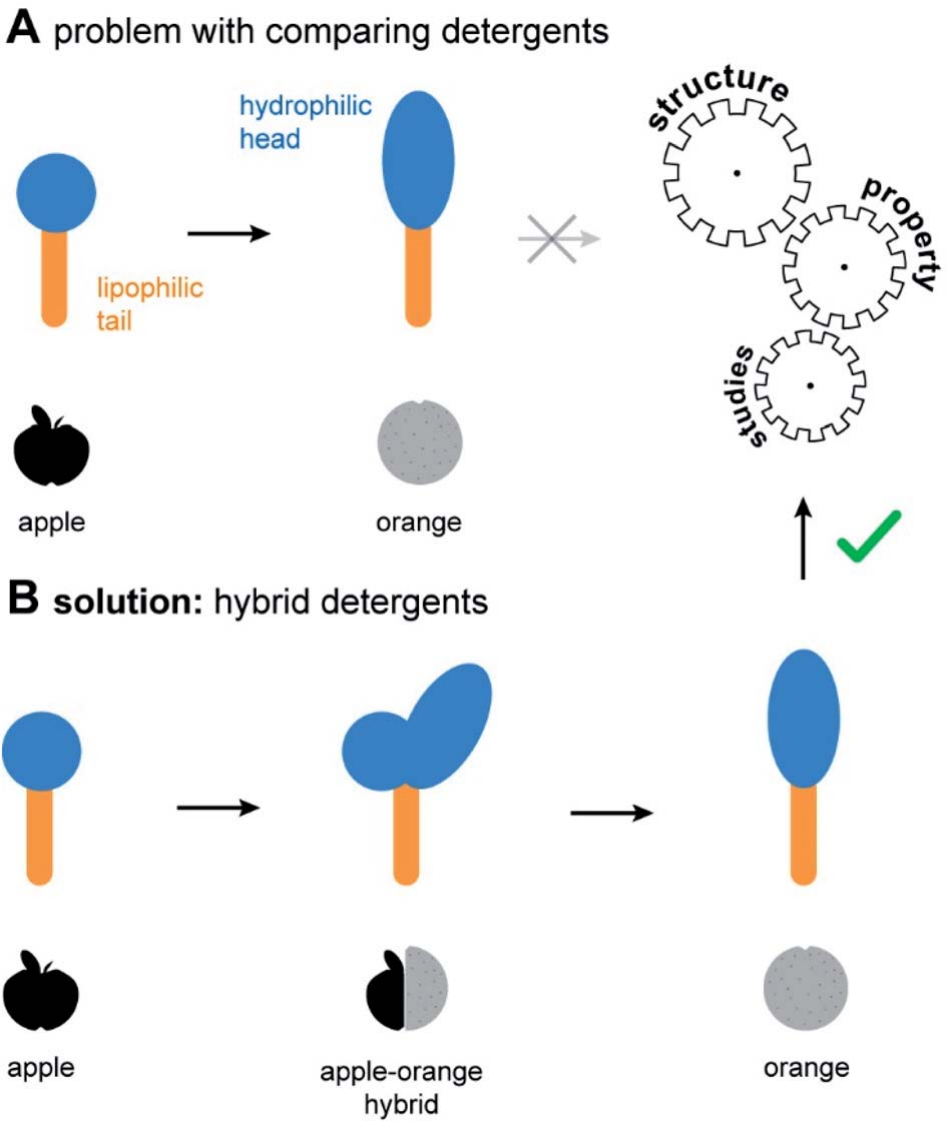
Overview of a common problem in detergent research. (A) Structurally different detergents can be difficult to compare, like apples and oranges. (B) Hybrid detergents provide the missing link in this comparison and will facilitate SPSs within the chemically diverse detergentome. Scalable hybrid detergents serve as the enabling step in the optimization of detergents for applications, including membrane protein research.

Non-ionic detergents are important in fundamental research, industry, and daily life.^1–5^ The molecular structure of non-ionic detergents consists of a hydrophilic head and a lipophilic tail (Fig. 1A). Due to their amphiphilic nature, detergents mediate the miscibility of hydrophilic and lipophilic substances. This forms the basis for various applications, such as cleaning, disinfection, emulsification, and drug discovery.^1–3,6^ Suitable detergents are identified by empirical screening, which drives up the time and costs of projects.^4,7^ To circumvent this problem, design guidelines are needed to allow for a predictable optimization of detergents. However, identifying design guidelines through structure–property studies (SPSs) is challenging. In comparative studies, often several structural detergent parameters are changed at once, thus rendering a conclusive SPS difficult (Fig. 1A).

Regardless of the application, the utility of detergents depends on their overall polarity and shape. To enable an efficient comparison of both properties among the diverse chemical space of the detergentome^8^ (entirety of all detergents), two concepts have been established: HLB^9^ and packing parameter.^10^ From an application-oriented point of view, packing parameters of detergents are currently important for the prediction of their aggregation behaviour in water.^10–12^ On the other hand, the HLB remains the metric of choice for the optimization of detergent formulations for industrial applications and daily life.^3^ However, currently available detergents do not cover the whole range of desired polarities and shapes, mainly due to a lack of efficient synthesis pathways. While the structure of the lipophilic tail plays a role in fine tuning of these properties, the polarity and shape of a detergent are mainly dependent on the structure of the hydrophilic head group.^12^ To enable a better fine tuning of detergent properties, expanding the chemical repertoire of detergent head groups is crucially needed.

Herein we expand the chemical space of the detergentome by establishing the combinatorial synthesis of hybrid detergents (Fig. 1B). Scalable hybrid detergents contain a combination of different detergent head groups and provide the missing link for the comparison of structurally different detergents. To exemplify utility, we apply the scalable hybrid detergents to address an interdisciplinary research challenge: The optimization of detergents in membrane protein research.

Even though the field of synthetic detergent chemistry recently surpassed its 100th anniversary,^1^ the predictable optimization of detergent molecules for applications in membrane protein research remains widely elusive.^4^ Membrane proteins are targets for 60% of current drugs,^13,14^ but a direct analysis of protein–drug interactions in biomembranes is often impossible.^6^ Biomembranes are heterogenous and not soluble in water. To circumvent these analytical challenges, a trick is applied: Similar to the cleaning of fatty dishes at home, non-polar membrane proteins are purified with detergents. Membrane proteins are encapsulated in a shell of detergent molecules. They become water-soluble and can be purified by chromatography.^8^ For decades, detergents are being routinely applied in membrane protein purification.^15^ However, it still remains puzzling how the molecular structure of detergents affects purification outcomes. Here, we harness the potential of scalable hybrid detergents to understand how detergent polarity and shape affect critical purification parameters, including protein yields and activity. We expect the impact of our work on fundamental detergent research and related disciplines to be as transformative as the introduction of scalable dendritic amphiphiles in the early 1990s.^16^

## Results and discussion

### Combinatorial synthesis

**Fig. 2:**
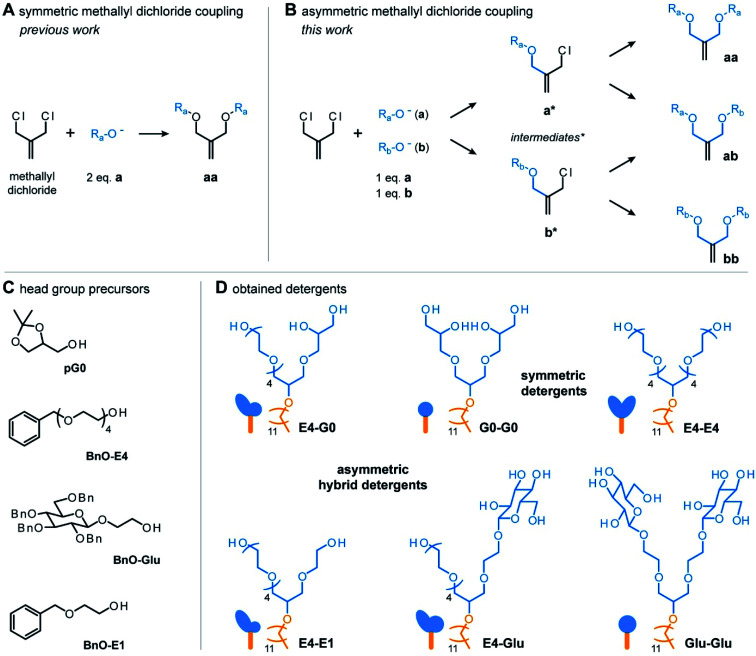
Overview of key steps and precursors for the combinatorial synthesis of hybrid detergents. (A) Schematic showing that a symmetric product is obtained when methallyl dichloride is reacted with two equivalents of the same nucleophile. (B) Schematic showing the steps involved in the reaction of methallyl dichloride with two different nucleophiles that lead to the obtainment of three different products. (C) Overview about head group precursors that enable the combinatorial synthesis of hybrid detergents shown in (D).

To enable the synthesis of hybrid detergents with scalable properties, we first expanded the chemical repertoire of detergent head groups. To do so, we harnessed the potential of a previously established coupling procedure.^17^ In this approach, methallyl dichloride is reacted with two equivalents of the same nucleophile (a) in tetrahydrofuran under basic conditions. Substitution of both chlorine atoms in methallyl dichloride by nucleophiles leads to the obtainment of symmetric products (aa) (Fig. 2A).^17^ The Haag group established this approach for the preparation of first-, second-, third-, fourth-, and fifth generation, acetal-protected oligoglycerol dendrons, *e.g.*, [pG1]–[pG5].^17^ These dendrons are the starting material for the preparation of homogenous oligoglycerol detergents (OGDs), which today have been established for various applications.^16^ On the other hand, Urner and co-workers found that reacting two different nucleophiles (a, b) with methallyl dichloride results in three different products (aa, ab, bb) (Fig. 2B).^4,18^ Mechanistically, it is anticipated that only one chlorine atom of methallyl dichloride is substituted at a time by either nucleophile a or b, and that the same applies for the formed intermediates (Fig. 2B).^18^

Following this approach, a mixture of two [pG1] regioisomers (a, b) was successfully converted to a mixture of three [pG2] regioisomers (aa, ab, bb).^4,18^ However, structurally similar regioisomers could not be separated by chromatography and were directly converted to a mixture of three [G2] OGD regioisomers.^4,18,19^

Encouraged by the demonstration that the one-pot reaction of different nucleophiles (a, b) with methallyl dichloride leads to three different molecules (aa, ab, bb),^18^ we translated this approach into a combinatorial synthesis to enable the preparation of three detergent head groups that (i) differ gradually in terms of polarity and shape, and (ii) can be separated by chromatography. Methallyl dichloride was reacted with the head group precursors of the established non-ionic detergents tetraethylene glycol monooctyl ether (C8E4) and [G1] OGD (G0-G0),^4^*e.g.*, tetraethylene glycol monobenzyl ether (BnO-E4) and solketal (G0) (Fig. 2C). In contrast to the structurally similar [G2] OGD regioisomers discussed before, the three products obtained from the reaction of methallyl dichloride with BnO-E4 and G0 could be readily separated by manual column purification on silica gel. Conveniently, the asymmetric hybrid detergent head group (ab) was the main product of the reaction, whereas symmetric head groups (aa, bb) were formed as by-products (Table S1†). Similar results were obtained from the reaction with BnO-E4 and the head group of *n*-octyl-β-d-glucoside (OG), *e.g.*, BnO-Glu (Table S1†). Similar results were also obtained from the combination of structurally more similar BnO-E4 and ethylene glycol monobenzyl ether (BnO-E1) (Table S1†). The obtained data highlight the robustness of this approach and show that combinatorial synthesis enables the preparation of three different detergent head groups in one step. Furthermore, combinatorial synthesis enables the straight-forward preparation of asymmetric hybrid head groups, which are more difficult to synthesize than symmetric ones.

**Fig. 3:**
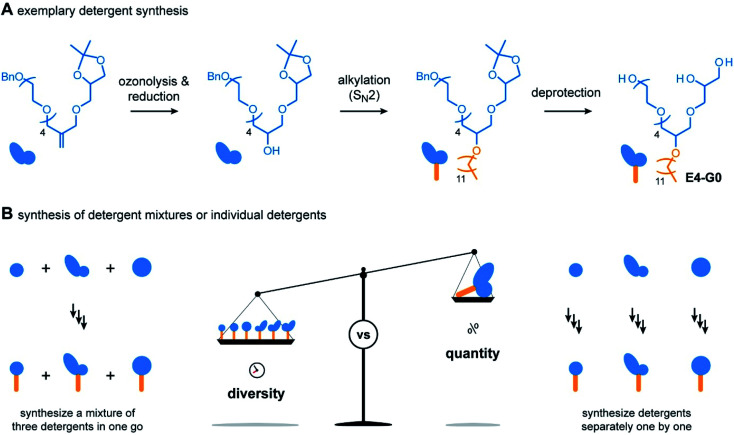
Overview about hybrid detergent synthesis and strategic options provided by the combinatorial synthesis. (A) Overview about the steps involved to accomplish the synthesis of detergents, namely ozonolysis and reduction, alkylation under S_N_2 conditions, and deprotection. (B) Schematic showing the two strategies provided by the combinatorial detergent synthesis. Detergent head group mixture obtained from the methallyl dichloride coupling can be converted to a detergent mixture, thus enabling the rapid preparation of chemically diverse detergent libraries (left). Alternatively, individual detergent head groups can be converted to detergents separately, allowing for up-scaling of the synthesis for individual detergents (right).

For the outcome of the combinatorial head group synthesis, it is important to protect all hydroxyl groups that are not involved in the reaction with methallyl dichloride. Ideal protecting groups for this purpose are stable against basic conditions and tolerate conditions that occur later in the detergent synthesis, such as oxidation with ozone, reduction with sodium borohydride, and alkylation under basic conditions (Fig. 3A).^4,17,18^ For 1,2-diols, the acetal-protecting group is currently most established and routinely used.^4,17,18^ Here, we used the benzyl protecting group as an alternative to also provide appropriate protection for individual hydroxyl groups, such as in the cases of BnO-E1, BnO-E4, and BnO-Glu (Fig. 2C). While benzyl groups are prone to oxidation in the presence of ozone, we recognized that a chemo-selective oxidation of double bonds is possible as long as the ozonolysis is done at −78° and prolonged saturation of the reaction with ozone is avoided.^20^

Having established the combinatorial synthesis of detergent head groups, we accomplished the synthesis of the detergents in two ways: (1) first, we followed the traditional synthesis approach in which individual head groups were isolated, alkylated, and subsequently deprotected (Fig. 3A).^18^ For this purpose, the double bonds of individual head groups were converted to hydroxyl groups by means of an established ozonolysis-reduction protocol.^17,18^ Subsequent alkylation of the hydroxyl groups and deprotection of the head groups under acidic and reductive conditions afforded individual detergents in gram scale.

When using the traditional synthesis approach, synthetic effort readily increases with the size of the detergent library. To accelerate the synthesis of detergent libraries, we also evaluated an alternative approach: (2) here, the trimeric head group mixtures obtained from the methallyl dichloride coupling into to trimeric detergent mixtures (Fig. 3B). The trimeric detergent mixtures were remarkably clean, as revealed by analytical HPLC (Fig. S1 and S2†). We conclude that the combinatorial synthesis provides an easy access to trimeric detergent mixtures, which opens new avenues for applications in which heterogeneity plays a key role, for example, protein purification,^4,18^ waste-water treatment,^21^ and mRNA delivery.^22,23^ However, more important for the present work is that individual detergents can be readily obtained upon HPLC purification in 100 mg scale. Taken together, both strategies (1) and (2) enable the synthesis of the detergents shown in Fig. 2D. However, none of them provides maximum efficiency in terms of purification and yields (Fig. 3B). Instead, both synthesis strategies (1) and (2) complement each other. The mixed synthesis strategy (2) accelerates the discovery of chemically diverse detergents, which is useful for screening applications aimed at the identification of new detergents. On the other hand, once optimal detergents have been identified, the first approach (1) enables the scale up of the synthesis. Therefore, we anticipate that the combinatorial detergent synthesis will accelerate the library-oriented discovery of custom-made detergents for various applications in the future.

### Scalable properties

**Fig. 4:**
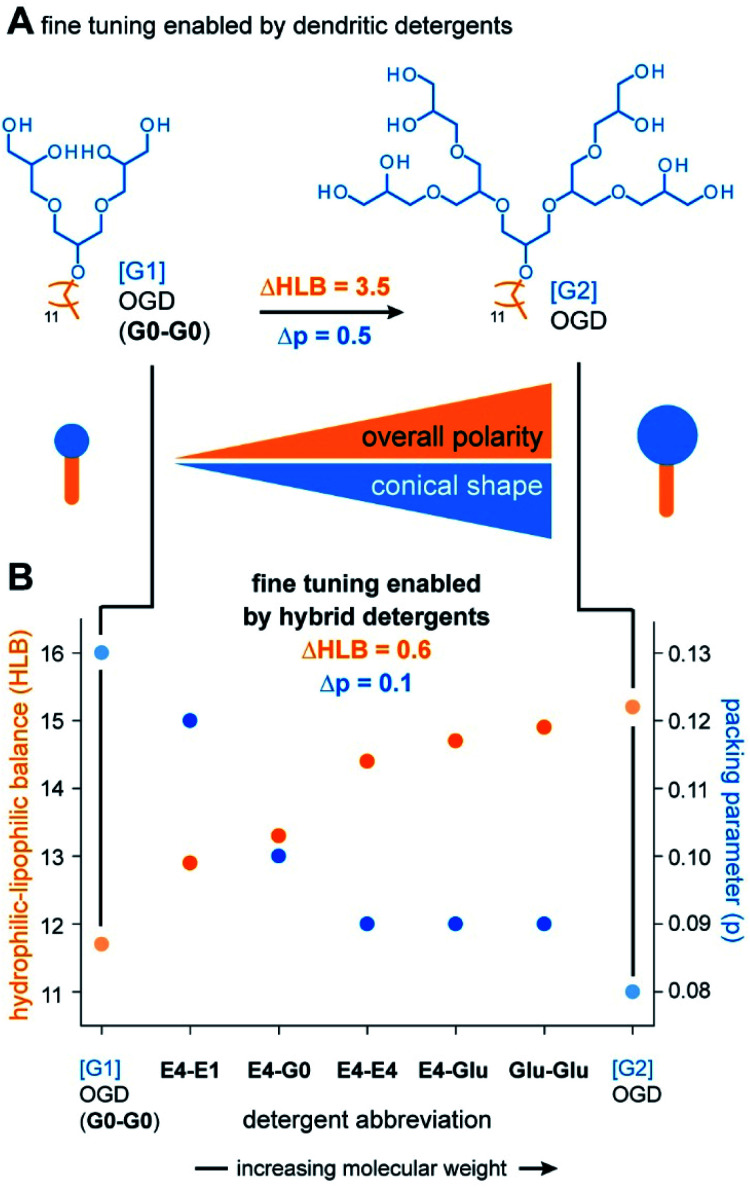
Property scaling enabled by hybrid detergents. (A) Rough tuning of polarity and shape is enabled by dendritic detergents as indicated by large HLB and packing parameter differences. (B) HLB and packing parameters of dendritic detergents and hybrid detergents. Overall polarity and conical shape of scalable hybrid detergents gradually increase with an unprecedented resolution in the direction from G0-G0 to Glu-Glu.

The most important and transformative result obtained from the combinatorial synthesis is the finding that three detergent head groups are obtained that gradually differ in terms of polarity and size. We expected that this result enables the preparation of detergent molecules that gradually differ in terms of polarity and shape (Fig. 4A and B). To monitor changes in polarity and shape, we calculated HLB values and packing parameters of the detergents and plotted them against the detergent abbreviations (Fig. 4B). In line with our expectations, HLB values gradually increase with the molecular weight of our detergent head groups (Fig. 4B). This indicates that overall polarity and conical shape of scalable hybrid detergent molecules gradually increase in the direction from G0-G0 to Glu-Glu (Fig. 4B).

Today, libraries of dendritic detergents are commonly synthesized to systematically tune polarity and shape of detergents. The HLB difference (ΔHLB) enabled by increasing the dendron head group from [G1] OGD (G0-G0) to [G2] OGD is 3.5 (Fig. 4A). Much better fine tuning is obtained from hybrid detergents as indicated by smaller ΔHLBs of about 0.6 (Table S2†) (Fig. 4B). The same is true for the shape, which gradually becomes more conical when the molecular weight of hybrid head groups is increased as indicated by a decrease in packing parameter (Table S3†) (Fig. 4B). We conclude that hybrid detergents enable a unique fine tuning of overall polarity and shape, which will facilitate the identification of new detergents with tailor-made polarities.

In summary, every detergent has individual characteristics and a detergent that is suitable for all applications does not exist. Nevertheless, in a recent pilot study, an asymmetric hybrid detergent was obtained by combining the head groups of *n*-dodecyl-β-d-maltoside (DDM) and C8E4 through a lipophilic hydroquinone scaffold.^8^ The comparative study revealed that hybrid detergents have the potential to combine beneficial properties of different detergents while neglecting some of their disadvantages. We expect hybrid detergents to not only widen the chemical space of the detergentome by expanding the chemical repertoire of detergent head groups, but to also bring us closer to the ability to design detergents that are more widely applicable. However, efforts towards the evaluation of the full potential of hybrid detergents through comprehensive SPSs were hampered by a lack of efficient synthesis procedures.^8^ Established protocols that enable a fusion of different detergent head groups into one scaffold are available from sequence-defined Janus glycodendrimers.^24^ However, compounds obtainable from these protocols include polyaromatic templates and triazole groups.^24^ Conclusive SPSs following these protocols are not possible. Established detergent molecules often do not contain triazole groups in combination with polyaromatic templates. Our combinatorial synthesis of hybrid detergents is bridging this gap by enabling (i) an efficient fusion of different head groups into hybrid detergent molecules while (ii) providing a comparable lipophilic tail. We anticipate our combinatorial synthesis approach will transform the optimization of detergents for challenging future applications.

### Membrane protein purification

The ability to systematically scale molecular properties of detergents through combinatorial synthesis offers new possibilities for SPSs in various research fields. A holy grail in fundamental detergent research is the ability to enable the purification of functionally active membrane proteins.^4,5,18,25^ Recent progresses in the development of screening platforms facilitate the assessment of detergents and detergent–lipid combinations for stabilizing membrane proteins in the absence of biomembranes.^26,27^ Although it is well-known that membrane protein activity varies between detergents and lipids, it is not well understood how one can rationally optimize the molecular structure of detergents. Today, widely applicable detergent selection rules help to narrow down the chemical space of the detergentome to be considered for screenings. For example, non-ionic detergents are more likely to enable the purification of functional membrane proteins than ionic detergents.^28,29^ More specific design guidelines are available from individual detergent classes, such as oligoglycerol detergents or saccharide detergents.^4,30^ These design guidelines link purification outcomes to detergent-class specific building block combinations, such as a specific oligoglycerol dendron generation in combination with a linear or lipid-like nonpolar tail,^4,6,18^ but are difficult to translate between detergent classes. The development of widely applicable detergent design guidelines has not yet been achieved. The problem: When comparing the utility of different detergent classes, more than one parameter changes at a time, for example, the detergent structure and the detergent concentration required in purification buffers. Experimentalists are facing situations like the comparison of apples and oranges (Fig. 1A).

**Fig. 5:**
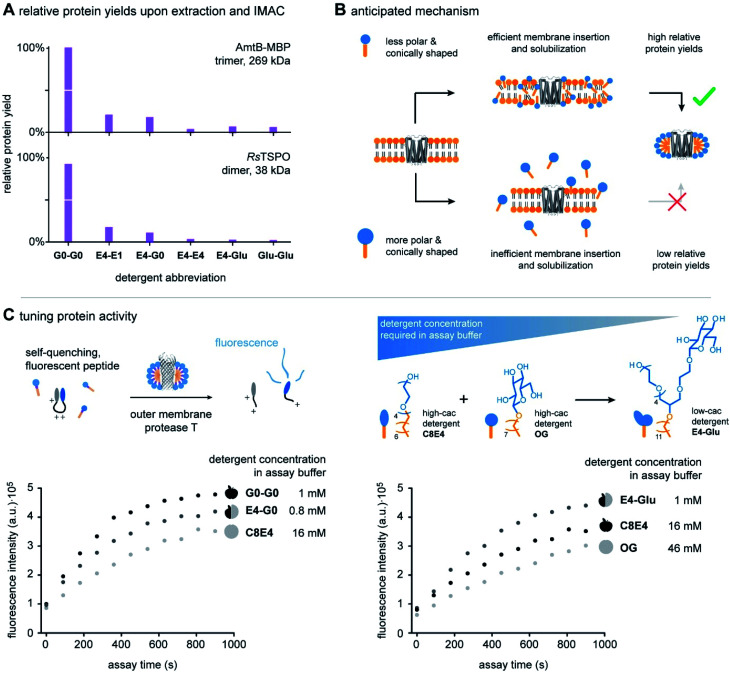
Utility of scalable hybrid detergents for protein purification. (A) Bar chart showing relative protein yields obtained from scalable hybrid detergents upon extraction and IMAC. Relative protein yields decrease from G0-G0 to Glu-Glu. (B) We anticipate that increasing the overall polarity and shape of detergents in the direction from G0-G0 to Glu-Glu leads to less efficient membrane insertion and solubilization. Therefore, lower relative protein yields are obtained. (C) Plots showing fluorescence intensity against assay time from the cleavage of a self-quenching fluorescent substrate by OmpT in different detergents and detergent concentrations. Protein activity increases when the detergent concentration is decreased. Combinatorial synthesis enables the production of low-cac detergents for the benefit of lower detergent concentrations in assay buffers and higher protein activities compared to high-cac detergents.

Here, we apply scalable hybrid detergents to investigate as to whether considering the overall polarity and shape can be used as widely applicable detergent design criterions to predict purification outcomes, including protein yields and activity. The first step in membrane protein purification is typically the extraction of proteins from membranes. To understand how polarity and shape of detergent molecules affect membrane protein extraction, we purified the integral membrane ammonia channel (AmtB) and translocator protein (*Rs*TSPO) from *E. coli* with scalable hybrid detergents under comparable conditions by following an established protocol.^4^ Briefly, membrane proteins were overexpressed in *E. coli* with a polyhistidine-tag and membrane fractions were isolated upon lysis, supernatant clarification, and ultracentrifugation. Protein-containing membranes were solubilized with detergents and purified by immobilized metal affinity chromatography (IMAC). Subsequently, relative protein yields were determined by UV/VIS spectroscopy and plotted against detergent abbreviations (Fig. 5A).

Relative protein yields decreased in the direction from the detergents G0-G0 to Glu-Glu (Fig. 5A). This indicates that increasing the relative polarity and conical shape of detergent molecules from G0-G0 to Glu-Glu decreases the detergents' utility for purifying large protein quantities from biological membranes. The extraction of large protein quantities can fail due to two reasons: (i) detergent molecules are not able to efficiently release proteins from membranes during extraction. (ii) Detergent molecules do not stabilize extracted membrane proteins in the aqueous phase. As a control experiment, we decoupled protein extraction from protein solubilization. Both proteins AmtB and RsTSPO were purified with a reference detergent DDM and transferred into scalable hybrid detergents by size-exclusion chromatography. Protein solutions obtained upon detergent exchange were stable over multiple freeze–thaw cycles. Furthermore, circular dichroism (CD) spectroscopy revealed that all scalable hybrid detergents can preserve the expected fold of the proteins in solution to similar degrees (Fig. S3 and S4†). Taken together, this indicates that increasing the relative polarity and conical shape of scalable hybrid detergent molecules decreases their ability to release proteins from membranes during extraction.

More broadly, our findings agree with findings from Umbreit and Strominger, who tested a broad range of different detergent classes and found that most suitable detergents for the solubilization of a membrane-bound carboxypeptidase have HLB values between 12 and 13.5.^31^ In a recent pilot study, a similar correlation between HLB and protein yields was obtained from the comparison of DDM, tetra ethylene glycol monooctyl ether (C8E4), and a DDM**-**C8E4 hybrid.^8^ Furthermore, the outcomes of both studies principally agree with results from Youn *et al.* who found that foldable, non-ionic saccharide detergents with HLBs between 11 and 13 can stabilize membrane proteins in solution in the absence of membranes.^32^ Considering this background, our data complement the state of the art by indicating that non-ionic detergent molecules with HLB values above 13.5 are less suitable for the extraction of membrane proteins than those with HLB values between 12 and 13.5.^31^ However, our data also show that non-ionic detergent molecules with HLB values above 13.5 can efficiently solubilize membrane proteins upon detergent exchange. Expanding the pool of non-ionic detergent molecules with HLBs beyond 13.5 provides new possibilities for membrane protein studies, because not every detergent that is good for protein extraction is equally compatible with biophysical techniques for the analysis of protein structure and function.^8^ Furthermore, scalable hybrid detergents complement the state of the art by bringing the molecular shape of detergent molecules into consideration (Fig. 4B). To rationalize purification outcomes, we expect that increasing the polarity and conical shape of non-ionic detergent molecules decreases their ability to insert into membranes (Fig. 5B). The uptake of detergent molecules into membranes is initializing membrane solubilization and key to the extraction of large protein quantities (Fig. 5B).^18,33^ Our data suggest that tuning the HLB and packing parameter of non-ionic detergent molecules beyond certain boundaries, *e.g.*, >13.5 in the case of the HLB and <0.1 in the case of the packing parameter, will lead to non-ionic detergent molecules that are more efficient in solubilizing proteins than in solubilizing membranes (Fig. 5B).

### Membrane protein activity

The structural and functional organisation of membrane proteins is commonly studied in detergent micelles.^6,25^ Protein activity is routinely optimized by changing various purification parameters, including buffer composition, purification time, temperature, detergents, or stabilizing ligands. Herein, we apply an established functional assay^4,18,34^ in which we keep all parameters constant, except for the detergent environment, to investigate how membrane protein activity can be optimized by tuning the design of detergent molecules.

To study protein activity, we purified the outer membrane protease T (OmpT)^35^ from *E. coli* with the lauryldimethylamine-*N*-oxide (LDAO) and exchanged the protein into scalable hybrid detergents. To prevent protein precipitation, the detergent concentrations were kept at two times of their critical aggregation concentration (cac). OmpT naturally acts as an aspartyl protease and is suggested to enhance the bacterial defence against antimicrobial peptides.^35,36^ The activity of OmpT depends on binding to lipopolysaccharide (LPS).^35^ To monitor activity, OmpT was incubated with LPS (*E. coli*, O111 : B4) and a self-quenching fluorescent substrate.^35^ Substrate cleavage increases sample fluorescence. Proteolytic activity can be monitored by fluorescence spectroscopy in a time-resolved manner (Fig. 5C).

To evaluate if protein activity can be rationalized by the polarity and shape of detergent molecules, we compared two sets of detergents: (1) hybrid detergent E4-G0 and the individual detergents G0-G0 and C8E4. (2) Hybrid detergent E4-Glu and the individual detergents OG and C8E4 (Fig. 5C). Interestingly, different activities were detected for all detergents. We found no evidence that any of the detergents is denaturing OmpT (Fig. S5†). Since the overall fold of OmpT is similar in all detergents, we hypothesized that protein activity is linked to the detergent concentration in assay buffers (Fig. 5C).

The detergent concentration applied in assay buffers is related to the cac, which in turn depends on the size, polarity, and shape of detergent molecules.^8,12^ Smaller, less polar, and less conically shaped detergent molecules, such as C8E4 and OG, are expected to require more detergent monomers to form a micelle than larger, more polar, and more conically shaped detergent molecules.^8,12^ Therefore, C8E4 and OG have larger cac values than G0-G0, E4-G0, and E4-Glu (Fig. 5C) (Table S4†). However, high-cac detergents interfere more efficiently with protein–lipid interaction than low-cac detergents.^8^ In other words, the high-cac detergents OG and C8E4 interfere more efficiently with OmpT-LPS binding than low-cac detergents G0-G0, E4-G0, and E4-Glu. Since binding of LPS to OmpT accelerates proteolytic activity, lower activities were obtained in the cases of OG and C8E4 (Fig. 5C).

Higher activities were also obtained from the low-cac detergents G0-G0 and E4-G0 compared to the high-cac detergent C8E4. However, closer analysis of the activities obtained from the low-cac detergents G0-G0 and E4-G0 indicate that the detergent concentration is not the only factor at play. Even though the cac of E4-G0 is slightly lower than the cac of G0-G0, a lower protein activity was obtained for E4-G0 (Fig. 5C) (Table S4†). HLBs and packing parameters indicate that the micelles formed by E4-G0 are less polar and less densely packed than the micelles formed by G0-G0. We speculate both detergents stabilize OmpT-LPS binding to varying degrees due to differences in polarity and packing of their micelles, which is reflected in different protein activities.

## Conclusion

In summary, we introduced the combinatorial synthesis of non-ionic, scalable hybrid detergents and demonstrated their utility in the context of membrane protein research. Combinatorial synthesis enables the choice between an accelerated production of diversified detergent libraries for small-scale applications or the large-scale production of individual detergents. Furthermore, HLB and packing parameter calculations revealed that combinatorial synthesis enables a gradual tuning of overall polarity and shape of hybrid detergent molecules. Membrane protein purification outcomes underlined that those detergent molecules with HLBs between 12 and 13.5 enable the extraction and solubilization of membrane proteins. Detergent molecules with HLBs beyond 13.5 may not be used for protein extraction but can solubilize membrane proteins upon detergent exchange. While the HLB concept enables estimating the overall polarity of detergent molecules, our study also puts the overall shape of detergents into consideration through the packing parameter concept. Our results indicate that both parameters, *i.e.*, polarity and shape, are determining factors for membrane protein studies. For example, increasing the overall polarity and conical shape of scalable hybrid detergent molecules decreases their ability to extract proteins from membranes. Furthermore, the ability to tune the overall polarity and shape of scalable hybrid detergent molecules facilitates the production of low-cac detergents for the benefit of protein activity. All aspects together create new possibilities for applications in membrane protein studies and beyond. Importantly, HLB and packing parameter concepts can theoretically be applied to any non-ionic detergent class and may be developed into widely applicable design guidelines for non-ionic detergents in the future. We anticipate combinatorial synthesis of scalable hybrid detergents will be widely used as enabling step for challenging applications in today's world.

## Data availability

The datasets supporting this article have been uploaded as part of the ESI.†

## Author contributions

The work was conceptualized by L. H. Urner. All authors contributed to the synthesis of hybrid detergents. Membrane protein experiments were done by L. H. Urner. The manuscript was written by L. H. Urner with input from all authors.

## Conflicts of interest

Freie Universität Berlin and University of Oxford filed a joint patent application related to the application of scalable hybrid detergents for the purification and structural analysis of membrane proteins (GB2106700.4). The authors declare no conflict of interest.

## Supplementary Material

SC-013-D2SC03130B-s001
