# Supplementary material for: Combinatorial synthesis enables scalable designer detergents for membrane protein studies
Source: Chem Sci. 2022 Aug 30;13(35):10299–307. doi: 10.1039/d2sc03130b (PMC9473536; doi:10.1039/d2sc03130b)
Supplement: SC-013-D2SC03130B-s001 [file SC-013-D2SC03130B-s001.pdf]

Electronic Supplementary Material (ESI) for Chemical Science.
This journal is © The Royal Society of Chemistry 2022

Supporting Information

Combinatorial synthesis enables scalable designer detergents for membrane protein studies

Leonhard H. Urner, Armin Ariamajd, and Alex Weikum

Table of contents

1. Experimental procedures ... 2
1.1 General remarks to synthesis ... 2
1.2 Building blocks for combinatorial head group synthesis ... 3
1.3 Combinatorial head group synthesis ... 5
1.4 Synthesis of Individual Detergents ... 8
1.5 Synthesis of detergent mixtures ... 15
1.6 Hydrophilic-lipophilic balance (HLB) calculation ... 18
1.7 Packing parameter calculation ... 18
1.8 Critical aggregation concentration ... 18
1.9 Circular dichroism spectroscopy ... 19
2. Supplementary Figures ... 20
3. Supplementary Tables ... 23
4. Literature ... 24

1. Experimental procedures

1.1 General remarks to synthesis

General information about chemicals, purification work flows, and compound characterization were adopted as previously described:[1] Chemicals were purchased from Sigma-Aldrich (Germany), Acros Organics (Germany), Alfa Aesar (Germany), Fluka (Germany), Fischer Scientific (Deutschland), Merk (Germany), TCI (Germany), and were used as supplied. Ethyl acetate (EtOAc) and (*n*-hexane) were distilled before they were used. Other solvents, such as methanol (MeOH), dimethylformamide (DMF), dichloromethane (DCM), and tetrahydrofuran (THF), were used as supplied. Dry solvents were purchased in bottles sealed with a septum or tapped from a solvent purification system (MS-SPS-800), which were brought from M. Braun (Germany). Deionized water (H_2O) used for synthesis was used from the tap and was provided by a deionization system installed in the Freie Universität's Institute of Chemistry and Biochemistry. Argon and oxygen were purchased from Linde (Germany) and used as supplied. For working under dry and oxygen-free reactions conditions, chemicals and solvents were handled under argon atmosphere. To support dry conditions the glassware was evacuated, heated up to 300 °C using a heat gun, and filled with argon prior usage. The ozone generator GIX8 was used (voltage: 6.3 V, current: 0.51 mA, oxygen volume flow: 22–25 mL/min) for ozonolysis reactions.

For reaction monitoring and purification procedures normal phase (NP) thin-layer chromatography (TLC) analysis was applied. NP TLC plates (DC-Fertigfolien ALUGRAM® Xtra SIL G/UV254) based on silica (SiO_2) were purchased from Macherey-Nagel (Germany). Silica gel (60 M) for preparative normal phase column chromatography was purchased from Macherey-Nagel. For NP TLC analysis and manual NP column purification mixtures of organic solvents (*v:v*) were prepared. If necessary, MeOH was added in percent per volume to the prepared mixtures (*v*:*v* + *v*%). TLC plates were either analyzed under UV irradiation (254 nm) using a lamp from CAMAG (Germany) or by staining the TLC plates either with cerium reagent (940 mL H_2O, 60 mL H_2SO_4, 25 g molybdic acid, 10 g cerium(IV) sulfate) or a potassium permanganate solution (250 mL H_2O, 2.5 g potassium permanganate). For the staining process, the TLC plates were fully submerged into the staining solution, excess of staining reagent was wiped off with cellulose, and the plate was heated up to 300 °C with a heat gun until staining was completed. Product mixtures, for example, **aa**:**ab**:**bb**, were quantified by ^{13}C NMR (inverse-gated mode). To determine the relative product proportions the relative intensities of the focal point signals were extracted from the NMR spectra. Sample concentrations were adjusted to 180 mg/mL.

Final detergent batches were purified by preparative reversed phase (RP) high-pressure liquid chromatography (HPLC). Raw products were dissolved in mixtures of deionized H_2O and MeOH (*v*:*v*, 1:1) and passed through an syringe filter (RC, 0.22 µM) prior to purification. The analytical HPLC system was equipped with two Smartline 1000 pumps, variable wavelength UV detector 2500, and an Autosampler 3950. As stationary phase a pre-packed Kinetex EVO C18 column was used (pore size: 100 Å, particle size: 5 µm, length: 250 mm, diameter: 4.6 mm), purchased from phenomenex (Germany). The preparative HPLC system was equipped with a LC-8A pump from Shimadzu (Germany), a UV variable wavelength monitor, and a differential refractometer from Knauer (Germany). As stationary phase a pre-packed Kinetex EVO C18 column was used (pore size: 100 Å, particle size: 5 µm, length: 250 mm, diameter: 21.2 mm), purchased from phenomenex (Germany). Degassed mixtures of H_2O and MeOH were used (*v*:*v*) and thermally equilibrated upon mixing for at least 16 h prior to use. Data processing and analysis was performed with an analog recorder L250E from Knauer. The HPLC systems were designed and constructed by Dr. Carlo Fasting (Freie Universität Berlin, Germany).

Mass spectra were acquired on an Agilent 6210 ESI-TOF (ESI-ToF) from Agilent Technologies (Santa Clara, CA, USA). The solvent flow rate was adjusted to 4 µL/min and the spray voltage was set to 4 kV. Drying gas flow rate was set to 15 psi (1 bar). All other parameters were adjusted for a maximum abundance of the relative $[M+H]^+$. The instrument was operated by the Core Facility BioSupraMol of the Freie Universität Berlin. ^1H NMR, ^{13}C NMR, and DEPT135 spectra were acquired using the following NMR instruments: Bruker DPX400 (^1H NMR: 400 MHz, ^{13}C NMR: 101 MHz), Jeol ECX400 (^1H NMR: 400 MHz, ^{13}C NMR: 101 MHz), Jeol ECP 500 (^1H NMR: 500 MHz, ^{13}C NMR: 126 MHz), Bruker

AVANCEIII500 (^{1}H NMR: 500 MHz, ^{13}C NMR: 126 MHz) or Bruker AVANCEIII700 (^{1}H NMR: 700 MHz, ^{13}C NMR: 175 MHz). All instruments were operated by the Core Facility BioSupraMol of the Freie Universität Berlin, too. Data processing and analysis was performed with MestReNova (*v*6.0.2-5475).

1.2 Building blocks for combinatorial head group synthesis

The following building blocks were used for the combinatorial head group synthesis: ethylene glycol monobenzyl ether (**BnO-E1**), tetraethylene glycol monobenzyl ether (**BnO-E4**), DL-1,2-Isopropylideneglycerol (**pG0**)**,** and a perbenzylated glucose derivative (**BnO-Glu**), Supplementary Scheme 1). Apart from the perbenzylated glucose derivative, all building blocks were purchased and used as supplied. The perbenzylated glucose derivative was synthesized by using the procedures described below (Supplementary Scheme 2).

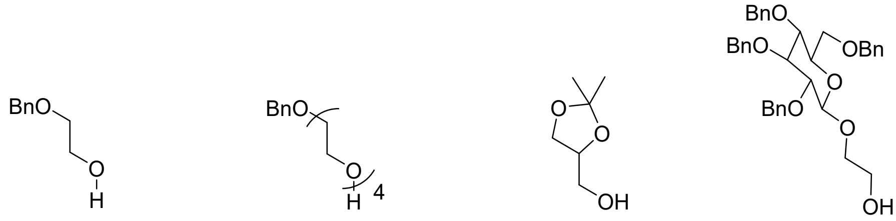

BnO-E1 **BnO-E4** **pG0** **BnO-Glu**

Supplementary Scheme 1. Utilized building blocks for the combinatorial synthesis of detergent head groups.

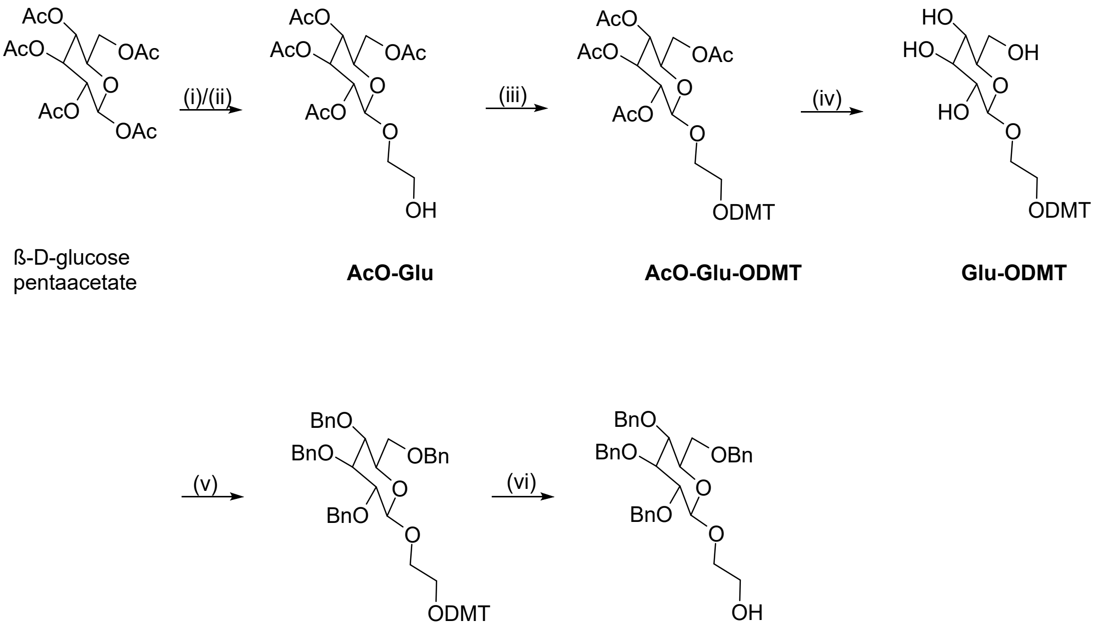

ß-D-glucose pentaacetate **AcO-Glu** **AcO-Glu-ODMT** **Glu-ODMT**

BnO-Glu-ODMT **BnO-Glu**

Supplementary Scheme 2. Synthesis of **BnO-Glu** was started from commercially available ß-D-glucose pentaacetate and achieved in six steps (i) – (vi). Reaction conditions of used for the individual steps were as follows: (i) **Bn-E1**, $BF_3{\cdot}(OEt)_2$, DCM, - 10 °C to RT, 16 h; (ii) H_2 (1 bar), Pd/C (cat.), MeOH, RT, 24 h; (iii) 4,4'-dimethoxytrityl chloride, NEt_3, toluene/DCM (*v*:*v,* 4:1), RT, 24 h; (iv) NaOMe (cat.), MeOH, RT, 20 h; (v) NaH (60w%), benzyl bromide, DMF, 50 °C, 20 h; (vi) HCl (37w%), MeOH/DCM (*v*:*v*, 16:1), RT, 20 h. Detailed information about the applied synthesis and purification procedures is given below.

Synthesis of AcO-Glu. ß-D-Glucose pentaacetate (20.0 g, 51.2 mmol) and **Bn-E1** (21.8 mL, 153 mmol) were dissolved in dry DCM (250 mL). The flask was cooled with an ice bath (-10 °C) and $BF_3·(OEt)_2$ (19.3 mL, 154 mmol) was added dropwise. The mixture was left stirring at RT overnight. $NaHCO_3$ (100 mL of a 0.60 M aqueous solution) was added in small portions and the mixture was left stirring at RT for 1 h. The organic phase was separated, washed with $NaHCO_3$ (3 x 100 mL of a 0.60 M aqueous solution), and dried over Na_2SO_4. Solids were filtered off, solvent was removed under reduced pressure, and the raw product was purified by column chromatography (SiO_2, DCM/EtOAc, 9:1). Solvent was removed under reduced pressure and the remaining material (28.7 g) was dissolved in MeOH (150 mL). Pd/C (Pd on carbon, loading 10w%, 2.15 g) and one drop of concentrated acetic acid were added. The mixture was stirred under hydrogen atmosphere (1 bar) at RT for 24 h. The suspension was passed through a syringe filter (RC, 0.22 µM). The solvent was removed under reduced pressure. Subsequent column chromatography (SiO_2, DCM/EtOC, 9:1 → 13:3 + 10% MeOH) gave the desired product as a white solid (9.70 g, 24.7 mmol, 48%). ^1H NMR (400 MHz, $CDCl_3$, ppm): δ = 5.18 (t, 1H), 5.05 – 4.95 (m, 2H), 4.52 (d, 1H), 4.19 – 4.12 (m, 2H), 3.84 – 3.77 (m, 2H), 3.74 – 3.63 (m, 3H), 2.48 (s, 1H), 2.06 (s, 3H), 2.02 (s, 3H), 1.99 (s, 3H), 1.97 (s, 3H). ^{13}C NMR (101 MHz, $CDCl_3$, ppm): δ = 170.7, 170.2, 169.5, 101.4, 73.1, 72.6, 71.9, 71.3, 68.4, 62.0, 61.9.

Synthesis of AcO-Glu-ODMT. The starting material **AcO-Glu** (12.0 g, 30.6 mmol) was dissolved in toluene (400 mL) and DCM (100 mL). After adding NEt_3 (5.93 mL, 42.8 mmol) and 4,4´-dimethoxytrityl chloride (12.4 g, 36.6 mmol), the reaction mixture was stirred at RT for 24 h. The solvent was removed under reduced pressure and the raw product was purified by column chromatography (SiO_2, DCM/EtOAc, 8:1 → 6:3 + 10 % MeOH)[1†] to obtain the desired product **AcO-Glu-ODMT** (19.2 g, 27.6 mmol, 90%). ^1H NMR (400 MHz, CD_3OD, ppm): δ = 7.45 – 7.40 (m, 2H), 7.33 – 7.23 (m, 6H), 7.21 – 7.14 (m, 1H), 6.86 – 6.80 (m, 4H), 5.29 (t, 1H), 5.09 – 4.98 (m, 2H), 4.76 (d, 1H), 4.30 – 4.25 (m, 1H), 4.14 – 4.07 (m, 1H), 3.96 – 3.90 (m, 1H), 3.87 – 3.82 (m, 1H), 3.76 (s, 6H), 3.72 – 3.65 (m, 1H), 3.29 – 3.26 (m, 1H), 3.11 – 3.04 (m, 1H), 2.01 (s, 6H), 1.98 (s, 3H), 1.89 (s, 3H). ^{13}C NMR (101 MHz, CD_3OD, ppm): δ = 172.3, 171.6, 171.2, 160.5, 146.6, 137.4, 137.2, 131.2, 129.3, 128.7, 127.7, 114.0, 101.9, 87.1, 74.3, 72.8, 70.6, 69.8, 63.8, 63.0.

Synthesis of Glu-ODMT. The compound **AcO-Glu-ODMT** (19.2 g, 27.6 mmol) was dissolved in MeOH (500 mL), catalytic amounts of sodium methoxide (1 mL of a 0.50 M solution in MeOH), and the mixture was stirred at RT for 20 h. The solvent was removed under reduced pressure. Subsequent column chromatography (SiO_2, DCM/EtOAc 4:1 → EtOAc + 10% MeOH)[1†] gave the desired product **Glu-ODMT** (12.1 g, 23.0 mmol, 83%)[1†] as a pale-yellow colored oil. ^1H NMR (400 MHz, CD_3OD, ppm): δ = 7.48 – 7.43 (m, 2H), 7.35 – 7.30 (m, 4H), 7.27 – 7.21 (m, 2H), 7.19 – 7.14 (m, 1H), 6.86 – 6.78 (m, 4H), 4.36 (d, 1H), 4.02 – 3.97 (m, 1H), 3.89 – 3.83 (m, 1H), 3.79 – 3.74 (m, 1H), 3.72 (s, 6H), 3.71 – 3.66 (m, 1H), 3.42 – 3.22 (m, 6H). ^{13}C NMR (101 MHz, CD_3OD, ppm): δ = 160.0, 159.9, 146.4, 137.3, 131.2, 129.3, 128.7, 127.7, 104.4, 87.4, 78.0, 77.8, 75.1, 71.5, 69.7, 64.2, 62.6, 55.6.

Synthesis of BnO-Glu-ODMT. The compound **Glu-ODMT** (12.1 g, 23.0 mmol) was dissolved in DMF (500 mL), NaH (60w%, 11.0 g, 275 mmol) was added in small portions, and the reaction mixture was stirred at RT for 2 h. Subsequently, benzyl bromide (28.0 mL, 234 mmol) was added dropwise, and the reaction mixture was stirred at 50 °C for 20 h. The reaction was allowed to cool down to RT and $NaHCO_3$ (300 mL of a 1.20 M aqueous solution) was slowly added. Organic solvent was removed under reduced pressure. The remaining material was suspended in H_2O (250 mL) and the aqueous layer was extracted with EtOAc (4 x 300 mL). The organic layers were washed with Brine (450 mL), H_2O (250 mL), and dried over Na_2SO_4. Solids were filtered off and the solvent was removed under reduced pressure. Column chromatography (SiO_2, pentate/EtOAc, 10:1 → 2:1)[2†] gave the desired product **BnO-Glu-ODMT** (14.3 g, 16.1 mmol, 70%). ^1H NMR (400 MHz, $(CD_3)_2CO$, ppm): δ = 7.55 – 7.51 (m, 2H), 7.42 – 7.16 (m, 27H), 6.87 – 6.81 (m, 4H), 5.12 (d, 1H), 4.95 (d, 1H), 4.87 – 4.77 (m, 3H), 4.65 – 4.53 (m, 4H), 4.09 – 4.03 (m, 1H), 3.86 – 3.51 (m, 12H), 3.47 – 3.41 (m, 1H), 3.38 – 3.27 (m, 2H). ^{13}C NMR (101 MHz, $(CD_3)_2CO$,

[1†] To pack the column, dry silica gel was suspended in DCM supplemented with 1v% NEt_3. Once the column was packed, solvent mixtures were used as described in the procedures above.
[2†] To pack the column, dry silica gel was suspended in *n*-pentane supplemented with 1v% NEt_3. Once the column was packed, solvent mixtures were used as described in the procedure above.

ppm): δ = 159.5, 146.2, 140.0, 139.6, 137.1, 137.0, 130.9, 129.0 – 128.0, 127.4, 113.8, 104.3, 86.7, 85.4, 83.2, 78.8, 75.9 – 74.9, 73.8, 69.9, 69.3, 63.8, 55.4.

Synthesis of BnO-Glu. The starting material **Glu-ODMT** (7.80 g, 8.79 mmol) was dissolved in MeOH (750 mL) and DCM (40 mL). HCl (600 µL of a 37w% aqueous solution) was added, and the mixture was stirred at RT for 20 h. Subsequently, the mixture was neutralized with NEt_3 (1.20 mL). Solvent was removed under reduced pressure. The remaining material was purified by column chromatography (SiO_2, DCM/EtOAc, 12:1 → 3:1) which led to the obtainment of **BnO-Glu** (3.28 g, 5.61 mmol, 64%). ^{1}H NMR (400 MHz, $(CD_3)_2CO$, ppm): δ = 7.42 – 7.22 (m, 20H), 5.01 (d, 1H), 4.93 (d, 1H), 4.85 – 4.79 (m, 2H), 4.72 (d, 1H), 4.64 – 4.52 (m, 4H), 3.95 – 3.89 (m, 1H), 3.81 – 3.53 (m, 8H), 3.39 – 3.35 (m, 1H), 2.83 (s, 1H). ^{13}C NMR (101 MHz, $(CD_3)_2CO$, ppm): δ = 139.6, 139.2, 139.1, 128.8 – 127.8, 104.4, 85.1, 82.9, 78.6, 75.7, 75.0, 74.8, 73.5, 72.6, 69.6, 62.0. MS(ESI+): *m/z* = 607.2676, $C_{36}H_{40}Na_1O_7^+$ (calculated = 607.2666).

1.3 Combinatorial head group synthesis

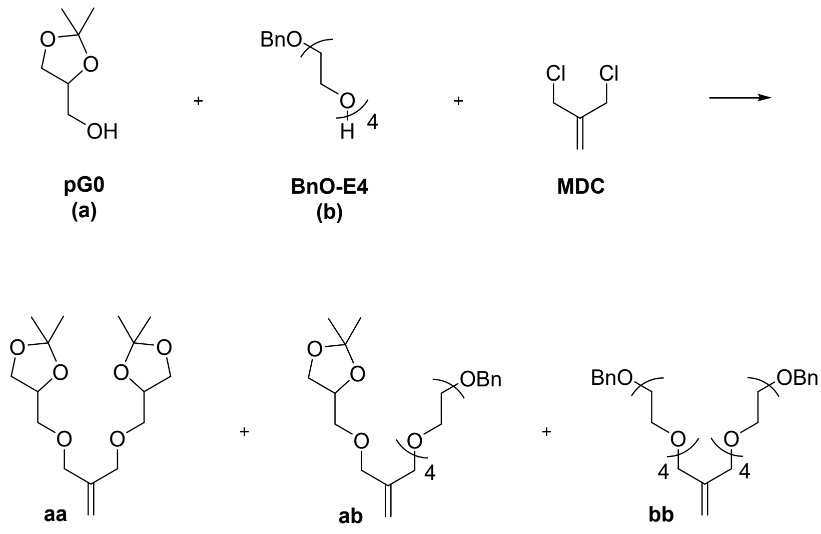

Supplementary Scheme 3. Combinatorial synthesis of three different head groups starting from **pG0** (**a**), **BnO-E4** (**b**), and methallyl dichloride (**MDC**). Detailed information about the applied synthesis and purification procedures is given below.

Combinatorial Head Group Synthesis (BnO-E4 + pG0). The starting material **BnO-E4** (5.00 g, 17.6 mmol) and **pG0** (2.36 g, 17.8 mmol) were dissolved in dry THF (300 mL). NaH (60w%, 2.14 g, 53.5 mmol) and catalytic amounts of 15-crown-5 were added, and the mixture was stirred at 50 °C for 1 h. Subsequently, **MDC** (2.02 mL, 17.5 mmol), catalytic amounts of 18-crown-6, and catalytic amounts of potassium iodide were added. The mixture was stirred at 80 °C for 25 h. The mixture was allowed to cool down to RT and H_2O (5 mL) was added. Solvent was removed under reduced pressure. The remaining material was suspended with a mixture of Brine (200 mL), H_2O (200 mL), and DCM (300 mL). The aqueous layer was extracted with DCM (3 x 300 mL). The combined organic layers were dried over Na_2SO_4, solids were filtered off, and solvent was removed under reduced pressure. Column chromatography (SiO_2, DCM/EtOAc, 1:0 → 1:1 + 4% MeOH) gave the desired products **aa** (220 mg, 0.70 mmol, 4%), **ab** (4.17 g, 8.90 mmol, 51%), and **bb** (2.41 g, 3.90 mmol, 22%). **Compound aa**: ^{1}H NMR (400 MHz, CD_3OD, ppm): δ = 5.21 – 5.18 (m, 2H), 4.30 – 4.22 (m, 2H), 4.08 – 4.02 (m, 6H), 3.76 – 3.70 (m, 2H), 3.52 – 3.43 (m, 4H), 1.40 – 1.31 (m, 12H). ^{13}C NMR (101 MHz, CD_3OD, ppm): δ = 144.1, 114.7, 110.4, 76.1, 72.8, 72.1, 67.5, 27.0, 25.6. MS(ESI+): *m/z* = 339.1802, $C_{16}H_{28}Na_1O_6^+$ (calculated = 339.1778). **Compound ab**: ^{1}H NMR (400 MHz, CD_3OD, ppm): δ = 7.38 – 7.25 (m, 5H), 5.20 – 5.18 (m, 2H), 4.55 (s, 2H), 4.28 – 4.22 (m, 1H), 4.07 – 4.02 (m, 5H), 3.76 – 3.61 (m, 15H), 3.59 – 3.55 (m, 2H), 3.52 – 3.42 (m, 2H), 1.39 - 1.32 (m, 6H). ^{13}C NMR (101 MHz, CD_3OD, ppm): δ = 144.2, 139.6, 129.3, 128.8, 128.6, 114.6, 110.4, 76.1, 74.1, 72.9, 72.5, 72.1, 71.5, 70.6, 67.5, 27.0, 25.6.

MS(ESI+): m/z = 491.2613, $C_{25}H_{40}Na_1O_8^+$ (calculated = 491.2615). **Compound bb**: ^{1}H NMR (400 MHz, CD_3OD, ppm): δ = 7.37 – 7.24 (m, 10H), 5.19 – 5.16 (m, 2H), 4.54 (s, 4H), 4.02 – 4.00 (m, 4H), 3.67 – 3.59 (m, 29H), 3.57 – 3.53 (m, 4H), 1.40 – 1.33 (m, 1H). ^{13}C NMR (101 MHz, $(CD_3)_2CO$, ppm): δ = 144.3, 139.6, 129.3, 128.8, 128.6, 114.4, 74.0, 72.5, 71.5, 70.63. MS(ESI+): m/z = 643.3444, $C_{34}H_{52}Na_1O_{10}^+$ (calculated = 643.3453).

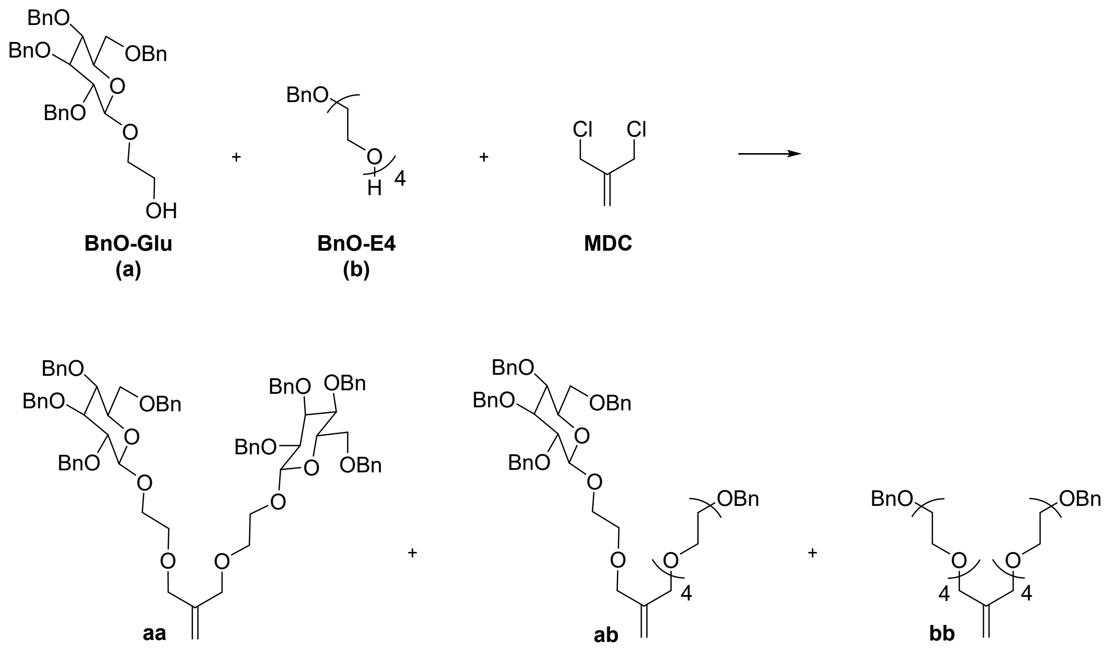

Supplementary Scheme 4. Combinatorial synthesis of three different head groups starting from **BnO-Glu** (**a**), **BnO-E4** (**b**), and methallyl dichloride (**MDC**). Detailed information about the applied synthesis and purification procedures is given below.

Combinatorial Head Group Synthesis (BnO-Glu + BnO-E4). The starting materials **BnO-E4** (2.92 g, 10.3 mmol) and **BnO-Glu** (6.00 g, 10.3 mmol) were dissolved in dry THF (250 mL). NaH (60w%, 1.23 g, 30.8 mmol) and catalytic amounts of 15-crown-5 were added and the mixture was stirred at 50 °C for 1 h. Subsequently, **MDC** (1.19 mL, 10.3 mmol), catalytic amounts of 18-crown-6, and catalytic amounts of potassium iodide were added. The mixture was stirred at 80 °C for 25 h. The mixture was allowed to cool down to RT and H_2O (10 mL) was added. Solvent was removed under reduced pressure. The remaining material was suspended with a mixture of Brine (200 mL), H_2O (150 mL), and DCM (400 mL). The aqueous layer was extracted with DCM (5 x 200 mL). The combined organic layers were dried over Na_2SO_4, solids were filtered off, and solvent was removed under reduced pressure. Column chromatography (SiO_2, DCM/EtOAc, 1:0 → 4:1 + 4% MeOH) gave the desired products **aa** (2.29 g, 1.88 mmol, 18%), **ab** (4.01 g, 4.35 mmol, 42%), and **bb** (0.84 g, 1.35 mmol, 13%). **Compound aa**: ^{1}H NMR (500 MHz, $(CD_3)_2CO$, ppm): δ = 7.46 – 7.18 (m, 40H), 5.16 (s, 2H), 5.02 (d, 2H), 4.94 (d, 2H), 4.84 (d, 2H), 4.80 (d, 2H), 4.72 (d, 2H), 4.66 – 4.51 (m, 8H), 4.12 – 3.94 (m, 6H), 3.89 – 3.48 (m, 16H), 3.38 (t, 2H). ^{13}C NMR (126 MHz, $(CD_3)_2CO$, ppm): δ = 144.6, 140.3, 140.2, 139.9, 139.8, 129.2, 129.1, 128.8, 128.6, 128.4, 128.3, 113.5, 104.6, 85.6, 83.3, 79.1, 76.1, 75.7, 75.5, 75.0, 73.9, 72.4, 70.4, 69.6. MS(ESI+): m/z = 1243.5718, $C_{76}H_{84}O_{14}Na_1^+$ (calculated = 1243.5753). **Compound ab**: ^{1}H NMR (500 MHz, $(CD_3)_2CO$, ppm): δ = 7.48 – 7.20 (m, 25H), 5.16 (s, 2H), 5.03 (d, 1H), 4.95 (d, 1H), 4.85 (d, 1H), 4.81 (d, 1H), 4.73 (d, 1H), 4.66 – 4.51 (m, 6H), 4.07 – 3.96 (m, 5H), 3.82 – 3.48 (m, 24H), 3.38 (t, 1H). ^{13}C NMR (126 MHz, $(CD_3)_2CO$, ppm): δ = 144.7, 140.3, 140.2, 140.1, 139.9, 139.8, 129.2, 129.1, 128.8, 128.6, 128.5, 128.4, 128.3, 113.3, 104.6, 85.6, 83.3, 79.1, 76.0, 75.7, 75.5, 75.0, 73.9, 73.6, 72.4, 72.3, 71.5, 71.4, 71.3, 70.6, 70.4, 70.2, 69.6. MS(ESI+): m/z = 943.4586, $C_{55}H_{68}O_{12}Na_1^+$ (calc. 943.4603). **Compound bb**: ^{1}H NMR (500 MHz, $(CD_3)_2CO$, ppm): δ = 7.40 – 7.25 (m, 10H), 5.15 (s, 2H), 4.54 (s, 4H), 4.01 – 3.98 (m, 4H), 3.68 – 3.48 (m, 32H). ^{13}C NMR (126 MHz, $(CD_3)_2CO$, ppm): δ =

144.8, 140.0, 129.1, 128.4, 128.2, 113.1, 73.5, 72.2, 71.4, 71.3, 70.6, 70.5. MS(ESI+): *m/z* = 643.3444, $C_{34}H_{52}Na_1O_{10}^+$ (calculated = 643.3453).

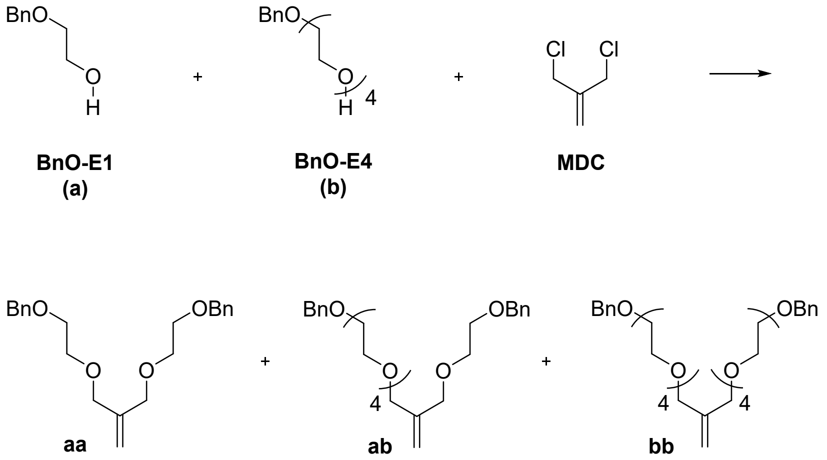

Supplementary Scheme 5. Combinatorial synthesis of three different head groups starting from **BnO-E1** (**a**), **BnO-E4** (**b**), and methallyl dichloride (**MDC**). Detailed information about the applied synthesis and purification procedures is given below.

Combinatorial Head Group Synthesis (BnO-E1+ BnO-E4). The starting material **BnO-E1** (2.68 g, 17.6 mmol) and **BnO-E4** (5.00 g, 17.6 mmol) were dissolved in dry THF (250 mL). NaH (60w%, 2.11 g, 52.7 mmol) and catalytic amounts of 15-crown-5 were added, and the mixture was stirred at 50 °C for 1 h. Subsequently, **MDC** (2.20 mL, 17.6 mmol), catalytic amounts of 18-crown-6, and catalytic amounts of potassium iodide were added. The mixture was stirred at 80 °C for 25 h. The mixture was allowed to cool down to RT and H_2O (10 mL) was added. Solvent was removed under reduced pressure. The remaining material was suspended with a mixture of Brine (150 mL), H_2O (150 mL), and DCM (400 mL). The aqueous layer was extracted with DCM (8 x 175 mL). The combined organic layers were dried over Na_2SO_4, solids were filtered off, and solvent was removed under reduced pressure. Column chromatography (SiO_2, DCM/EtOAc, 1:0 → 4:1 + 10% MeOH) gave the desired products **aa** (2.92 g, 4.70 mmol, 27%), **ab** (3.22 g, 6.59 mmol, 37%), and **bb** (1.21 g, 3.36 mmol, 19%). **Compound aa**: ^{1}H NMR (500 MHz, $CDCl_3$, ppm): δ = 7.33 – 7.14 (m, 10H), 5.13 (s, 2H), 4.49 (s, 4H), 3.97 (s, 4H), 3.61 – 3.47 (m, 8H). ^{13}C NMR (126 MHz, $CDCl_3$, ppm): δ = 142.7, 138.4, 128.5, 127.8, 127.7, 114.1, 73.3, 72.0, 69.7, 69.5. MS(ESI+): *m/z* = 379.1867, $C_{22}H_{28}O_4Na^+$ (calculated = 379.1885). **Compound ab**: ^{1}H NMR (500 MHz, $CDCl_3$, ppm): δ = 7.34 – 7.14 (m, 10H), 5.12 (s, 2H), 4.50 (s, 2H), 4.49 (s, 2H), 3.96 (s, 2H), 3.95 (s, 2H), 3.66 – 3.44 (m, 20H). ^{13}C NMR (126 MHz, $CDCl_3$, ppm): δ = 142.6, 138.4, 128.4, 127.8, 127.7, 114.1, 73.3, 71.9, 70.7, 69.7, 69.6, 69.5. MS(ESI+): *m/z* = 511.2678, $C_{28}H_{40}O_7Na^+$ (calculated = 511.2672). **Compound bb**: ^{1}H NMR (500 MHz, $(CD_3)_2CO$, ppm): δ = 7.40 – 7.25 (m, 10H), 5.15 (s, 2H), 4.54 (s, 4H), 4.01 – 3.98 (m, 4H), 3.68 – 3.48 (m, 32H). ^{13}C NMR (126 MHz, $(CD_3)_2CO$, ppm): δ = 144.8, 140.0, 129.1, 128.4, 128.2, 113.1, 73.5, 72.2, 71.4, 71.3, 70.6, 70.5. MS(ESI+): *m/z* = 643.3444, $C_{34}H_{52}Na_1O_{10}^+$ (calculated = 643.3453).

1.4 Synthesis of Individual Detergents

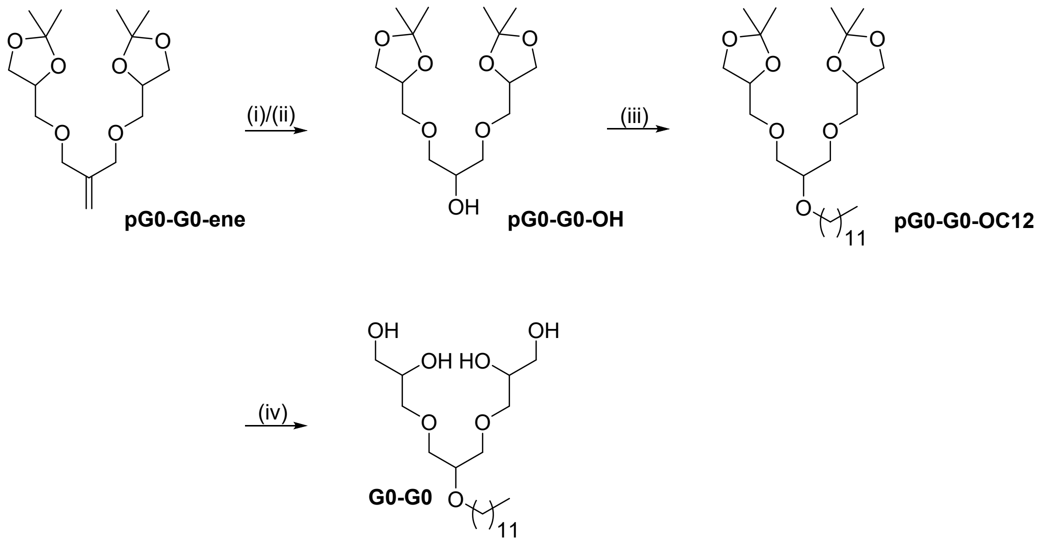

Supplementary Scheme 6. Synthesis of the individual detergent **G0-G0** was achieved in four steps. Reaction conditions were as follows: (i) O_3, DCM/MeOH (*v*:*v*, 1:1), -78 °C, 1h; (ii) $NaBH_4$, -78 °C to RT, 16 h; (iii) 1-bromododecane, NaH (60w%), DMF, 50 °C to RT, 17 h; (iv) HCl (37w%), MeOH, RT, 2 x 12 h. Detailed information about the applied synthesis and purification procedures is given below.

Synthesis of pG0-G0-OH. The starting material **pG0-G0-ene** (10.0 g, 31.6 mmol) was dissolved in dry MeOH (100 mL) and dry DCM (100 mL). The mixture was cooled down to - 78 °C and ozone was passed through it until its color changed to deep blue. Oxygen was then passed through the solution until it became colorless. Sodium borohydride (11.9 g, 316 mmol) was added, and the mixture was stirred and allowed to warm up to RT overnight. NH_4Cl (100 mL of a saturated aqueous solution) was added, and the mixture was stirred for 20 minutes. Organic solvent was removed under reduced pressure and the remaining material was suspended in H_2O (100 mL), and DCM (100 mL). The aqueous layer was extracted with DCM (2 x 40 mL). The combined organic layers were dried over Na_2SO_4, solids were filtered off, and solvent was removed under reduced pressure. Subsequent column chromatography (SiO_2, DCM/EtOAc 4:1 → 4:1 + 2% MeOH) led to the obtainment of **pG0-G0-OH** (9.43 g, 29.4 mmol, 93%). ^{1}H NMR (400 MHz, CD_3OD, ppm): δ = 4.29 – 4.23 (m, 2H), 4.07 – 4.03 (m, 2H), 3.89 – 3.83 (m, 1H), 3.75 – 3.71 (m, 2H), 3.59 – 3.46 (m, 8H), 1.41 – 1.31 (m, 12H). ^{13}C NMR (101 MHz, CD_3OD, ppm): δ = 110.4, 76.1, 73.9, 73.4, 70.5, 67.5, 27.0, 25.6. MS(ESI+): *m/z* = 343.1739, $C_{15}H_{28}Na_1O_7^+$ (calculated = 343.1727).

Synthesis of pG0-G0-OC12. The compound **pG0-G0-OH** (9.43 g, 29.4 mmol) was dissolved in DMF (200 mL) and NaH (60w%, 3.53 g, 88.3 mmol) was added in small portions. The mixture was stirred at 50 °C for 1 h. 1-Bromododecane (14.1 mL, 58.8 mmol) was added dropwise, and the mixture was stirred at RT for 16 h. H_2O (15 mL) was added drop wise and solvent was removed under reduced pressure. The remaining material was suspended in H_2O (100 mL), Brine (100 mL), and EtOAc (100 mL). The aqueous layer was extracted with EtOAc (4 x 50 mL). The combined organic layers were dried over Na_2SO_4, solids were filtered off, and solvent was removed under reduced pressure. Subsequent column chromatography (SiO_2, DCM/EtOAc 4:1 → 4:1 + 3% MeOH) led to the obtainment of **pG0-G0-OC12** (11.0 g, 22.5 mmol, 77%). ^{1}H NMR (400 MHz, CD_3OD, ppm): δ = 4.28 – 4.20 (m, 2H), 4.06 – 4.02 (m, 2H), 3.75 – 3.71 (m, 2H), 3.64 – 3.45 (m, 11H), 1.60 – 1.51 (m, 2H), 1.41 – 1.25 (m, 39H), 0.92 – 0.89 (m, 3H). ^{13}C NMR (101 MHz, CD_3OD, ppm): δ = 101.4, 79.1, 76.1, 73.3, 72.4, 72.3, 71.4, 67.6, 33.0, 31.1, 30.8, 30.7, 30.6, 30.5, 27.2, 27.1, 25.7, 23.7, 14.5. MS(ESI+): *m/z* = 511.3635, $C_{27}H_{52}Na_1O_7^+$ (calculate = 511.3605).

Synthesis of G0-G0. The starting material **pG0-G0-OC12** (11.0 g, 22.5 mmol) was dissolved in MeOH (1.5 L) and HCL (200 µL of a 37w% aqueous solution) was added. The mixture was stirred at RT for 12 h. Solvent was removed under reduced pressure and the procedure was repeated. Subsequent purification by RP HPLC purification (RPC18, H_2O:MeOH, *v*:*v*, 3:7, flow rate: 20 mL/min) led to the obtainment of **G0-G0** (5.30 g, 12.9 mmol, 58%). ^{1}H NMR (500 MHz, CD_3OD, ppm): δ = 3.78 – 3.73 (m, 2H), 3.64 – 3.44 (m, 15H), 1.59 – 1.54 (m, 2H), 1.41 – 1.24 (m, 18H), 0.92 – 0.89 (m, 3H). ^{13}C NMR (126 MHz, CD_3OD, ppm): δ = 79.1, 73.9, 72.2, 72.1, 71.5, 64.4, 33.0, 31.1, 30.7, 30.6, 30.4, 27.1, 23.7, 14.4. MS(ESI+): *m/z* = 431.3010 $C_{21}H_{44}Na_1O_7^+$ (calculate = 431.2979).

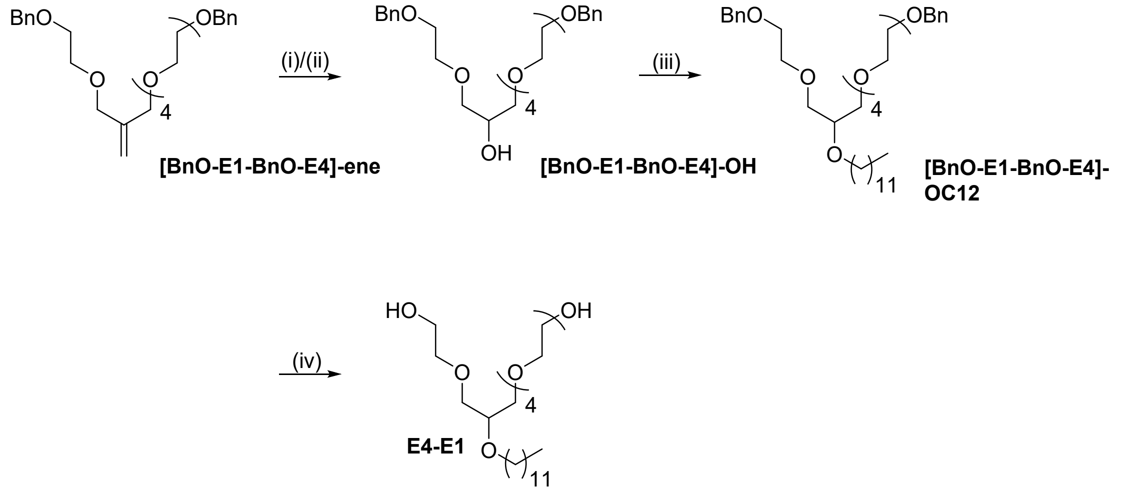

Supplementary Scheme 7. Synthesis of the individual detergent **E4-E1** was achieved in four steps. Reaction conditions were as follows: (i) O_3, DCM/MeOH (*v*:*v*, 1:1), -78 °C, 1h; (ii) $NaBH_4$, -78 °C to RT, 16 h; (iii) 1-bromododecane, NaH (60w%), DMF, 50 °C, 17 h; (iv) H_2 (1 bar), Pd/C (cat.), MeOH/THF (*v*:*v*, 10:1), RT, 48 h. Detailed information about the applied synthesis and purification procedures is given below.

Synthesis of [BnO-E1-BnO-E4]-OH. The starting material **[BnO-E1-BnO-E4]-ene** (3.22 g, 6.59 mmol) was dissolved in dry MeOH (40 mL) and dry DCM (40 mL). The mixture was cooled down to - 78 °C and ozone was passed through it until its color changed to deep blue. Oxygen was then passed through the solution until it became colorless. Sodium borohydride (2.49 g, 65.9 mmol) was added, and the mixture was stirred and allowed to warm up to RT overnight. NH_4Cl (5 mL of a saturated aqueous solution) was added, and the mixture was stirred for 1 h. Organic solvent was removed under reduced pressure and the remaining material was suspended in Brine (100 mL), H_2O (150 mL), and DCM (150 mL). The aqueous layer was extracted with DCM (8 x 200 mL). The combined organic layers were dried over Na_2SO_4, solids were filtered off, and solvent was removed under reduced pressure. Subsequent column chromatography (SiO_2, DCM/EtOAc 4:1 → 13:3 + 10% MeOH) led to the obtainment of **[BnO-E1-BnO-E4]-OH** (3.09 g, 6.27 mmol, 95%). ^{1}H NMR (400 MHz, $CDCl_3$, ppm): δ = 7.30 – 7.16 (m, 10H), 4.49 (s, 4H), 3.90 (quint, 1H), 3.66 – 3.37 (m, 24H), 2.92 (s, 1H). ^{13}C NMR (101 MHz, $CDCl_3$, ppm): δ = 138.3, 138.2, 128.5, 128.4, 127.8, 127.7, 73.3, 72.6, 72.5, 70.9, 70.8, 70.7, 70.6, 69.5, 69.4. MS(ESI+): *m/z* = 515.2625, $C_{27}H_{40}Na_1O_8^+$ (calculated = 515.2621).

Synthesis of [BnO-E1-BnO-E4]-OC12. The starting material **[BnO-E1-BnO-E4]-OH** (3.09 g, 6.27 mmol) was dissolved in DMF (100 mL). NaH (60w%, 0.75 g, 18.8 mmol) was added and the mixture was stirred at 50 °C for 90 minutes. 1-Bromododecane (3.01 mL, 12.6 mmol) was added and the mixture was stirred at 50 °C for 16 h. H_2O (3 mL) was added and the solvent was removed under reduced pressure. The remaining material was suspended in Brine (100 mL), H_2O (50 mL), and EtOAc (100 mL). The aqueous layer was extracted with EtOAc (8 x 200 mL). The combined organic layers were dried over Na_2SO_4, solids were filtered off, and solvent was removed under reduced pressure. Column chromatography (SiO_2, DCM/EtOAc, 14:1 → 2:1) led to the obtainment of the product **[BnO-E1-BnO-**

E4]-OC12 (3.45 g, 5.22 mmol, 83%). ^{1}H NMR (400 MHz, $CDCl_3$, ppm): δ = 7.30 – 7.16 (m, 10H), 4.48 (s, 4H), 3.62 – 3.42 (m, 25H), 1.52 – 1.43 (m, 2H), 1.29 – 1.11 (m, 20H), 0.80 (t, 3H). ^{13}C NMR (101 MHz, $CDCl_3$, ppm): δ = 138.4, 138.3, 128.4, 127.7, 127.6, 77.9, 73.3, 71.4, 71.3, 71.0, 70.9, 70.7, 70.6, 69.5, 32.0, 30.2, 29.7, 29.6, 29.4, 26.1, 22.7, 14.2. MS(ESI+): *m/z* = 699.4236, $C_{39}H_{64}K_1O_8^+$ (calculated = 699.4238).

Synthesis of E4-E1. The compound **[BnO-E1-BnO-E4]-OC12** (3.45 g, 5.22 mmol) was dissolved in MeOH (50 mL) and THF (5 mL). Pd/C (Pd on carbon, 10w% loading, 300 mg) was added and the mixture was stirred under hydrogen atmosphere (1 bar) at RT for 48 h. The mixture was passed through a syringe filter (0.22 µm, RC) and the solvent was removed under reduced pressure. RP HPLC purification (RPC18, H_2O:MeOH, *v*:*v*, 3:7, flow rate: 20 mL/min) led to the obtainment of detergent **E4-E1** (2.44 g, 5.08 mmol, 97%). ^{1}H NMR (500 MHz, CD_3OD, ppm): δ = 3.78 – 3.52 (m, 25H), 1.61 (m, 2H), 1.47 – 1.28 (m, 20H), 0.96 (m, 3H). ^{13}C NMR (126 MHz, CD_3OD, ppm): δ = 79.2, 74.0, 73.7, 72.0, 71.9, 71.6, 71.5, 71.4, 62.2, 33.1, 31.1, 30.8, 30.6, 30.5, 27.2, 23.7, 14.5. MS(ESI+): *m/z* = 503.3556, $C_{25}H_{52}Na_1O_8^+$ (calculated = 503.3560).

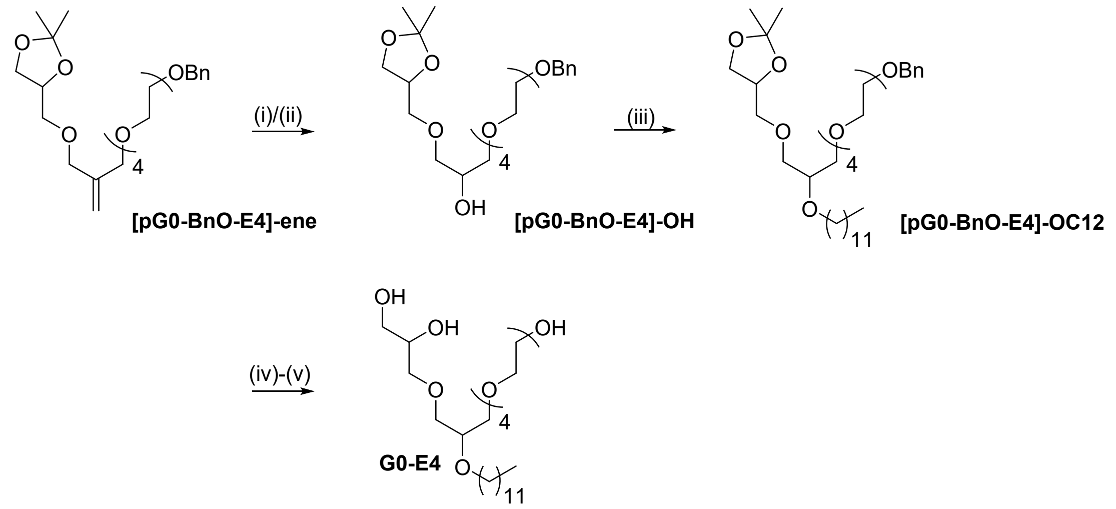

Supplementary Scheme 8. Synthesis of the individual detergent **G0-E4** was achieved in five steps. Reaction conditions were as follows: (i) O_3, DCM/MeOH (*v*:*v*, 1:1), -78 °C, 1h; (ii) $NaBH_4$, -78 °C to RT, 16 h; (iii) 1-bromododecane, NaH (60w%), DMF, 50 °C, 22 h; (iv) H_2 (5 bar), Pd/C (cat.), MeOH, RT, 15 h; (v) HCl (37w%), RT, 12 h.

Synthesis of [pG0-BnO-E4]-OH. The compound **[pG0-BnO-E4]-ene** (4.17 g, 8.90 mmol) was dissolved in dry MeOH (35 mL) and dry DCM (35 mL). The mixture was cooled down to - 78 °C and ozone was passed through it until its color changed to deep blue. Oxygen was then passed through the solution until it became colorless. Sodium borohydride (3.37 g, 89.1 mmol) was added, and the mixture was stirred and allowed to warm up to RT overnight. NH_4Cl (50 mL of a saturated aqueous solution) was added, and the mixture was stirred for 20 minutes. Organic solvent was removed under reduced pressure and the remaining material was suspended in H_2O (50 mL), and DCM (50 mL). The aqueous layer was extracted with DCM (2 x 40 mL). The combined organic layers were dried over Na_2SO_4, solids were filtered off, and solvent was removed under reduced pressure. Subsequent column chromatography (SiO_2, DCM/EtOAc 4:1 → 4:1 + 12% MeOH) led to the obtainment of the desired compound **[pG0-BnO-E4]-OH** (3.90 g, 8.30 mmol, 93%). ^{1}H NMR (400 MHz, CD_3OD, ppm): δ = 7.41 – 7.22 (m, 5H), 4.28 – 4.22 (m, 1H), 4.07 – 4.01 (m, 1H), 3.89 – 3.83 (m, 1H), 3.75 – 3.70 (m, 1H), 3.68 – 3.58 (m, 16H), 3.56 – 3.43 (m, 6H), 1.40 – 1.30 (m, 6H). ^{13}C NMR (101 MHz, CD_3OD, ppm): δ = 139.6, 129.3, 128.8, 128.6, 110.4, 76.1, 74.0, 73.6, 73.4, 71.7, 71.5, 70.6, 70.5, 67., 27.0, 25.6. MS(ESI+): *m/z* = 495.2575, $C_{24}H_{40}Na_1O_9^+$ (calculated = 495.2565).

Synthesis of [pG0-BnO-E4]-OC12. The starting material **[pG0-BnO-E4]-OH** (3.90 g, 8.30 mmol) was dissolved in DMF (100 mL). NaH (60w%, 0.97 g, 24.2 mmol) was added and the mixture was stirred at

50 °C for 3 h. 1-Bromododecane (3.88 mL, 4.03 g) was added, and the mixture was stirred at RT for 19 h. H_2O (10 mL) was added dropwise, and the solvent was removed under reduced pressure. The remaining material was suspended in Brine (100 mL), H_2O (100 mL), and EtOAc (100 mL). The aqueous layer was extracted with EtOAc (4 x 50 mL). The combined organic layers were dried over Na_2SO_4, solids were filtered off, and solvent was removed under reduced pressure. Column chromatography (SiO_2, DCM/EtOAc, 4:1 → 4:1 + 2% MeOH) gave the desired compound **[pG0-BnO-E4]-OC12** (3.92 g, 6.10 mmol, 73%). ^{1}H NMR (400 MHz, CD_3OD, ppm): δ = 7.37 – 7.24 (m, 5H), 4.55 (s, 2H), 4,27 – 4.19 (m, 1H), 4.06 – 4.00 (m, 1H), 3.75 – 3.69 (m, 1H), 3.68 – 3.46 (m, 25H), 1.59 – 1.50 (m, 2H), 1.40 – 1.23 (m, 24H), 0.93 – 0.86 (m, 3H). ^{13}C NMR (101 MHz, CD_3OD, ppm): δ = 139.6, 129.3, 128.8, 128.6, 110.4, 79.1, 76.1, 74.1, 73.3, 72.4, 71.9, 71.5, 71.4, 70.6, 67.6, 33.0, 31.1, 30.8, 30.7, 30.6, 30.5, 27.2, 27.1, 25.7, 23.7, 14.5. MS(ESI+): *m/z* = 663.4420, $C_{36}H_{64}Na_1O_9^+$ (calculated = 663.4443).

Synthesis of G0-E4. The compound **[pG0-BnO-E4]-OC12** (3.92 g, 6.10 mmol) was dissolved in MeOH (16 mL) and Pd/C (Pd on carbon, 10w% loading, 300 mg) was added. The mixture was stirred at RT under hydrogen atmosphere (5 bar) for 15 h. The mixture was passed through a syringe filter (0.45 µm, RC) and solvent was removed under reduced pressure. The remaining material was dissolved in MeOH (70 mL) and HCl (70.0 µL of a 37w% aqueous solution) was added. The mixture was stirred at RT for 12 h. NEt_3 (70.0 µL) was added, and the solvent was removed under reduced pressure. RP HPLC purification (RPC18, H_2O:MeOH, *v*:*v*, 3:7, flow rate: 20 mL/min) led to the obtainment of the detergent **G0-E4** (2.95 g, 5.10 mmol, 84%). ^{1}H NMR (500 MHz, CD_3OD, ppm): δ = 3.80 – 3.74 (m, 1H), 3.69 – 3.47 (m, 28H), 1.61 – 1.54 (m, 2H), 1.39 – 1.28 (m, 18H), 0.95 –0.89 (m, 3H). ^{13}C NMR (126 MHz, CD_3OD, ppm): δ = 79.2, 79.1, 73.9, 73.6, 72.2, 72.1, 72.0, 71.8, 71.6, 71.5, 71.4, 64.4, 62.2, 33.0, 30.7, 30.6, 30.4, 23.7. MS(ESI+): *m/z* = 533.3687, $C_{26}H_{54}Na_1O_9^+$ (calculated = 533.3660).

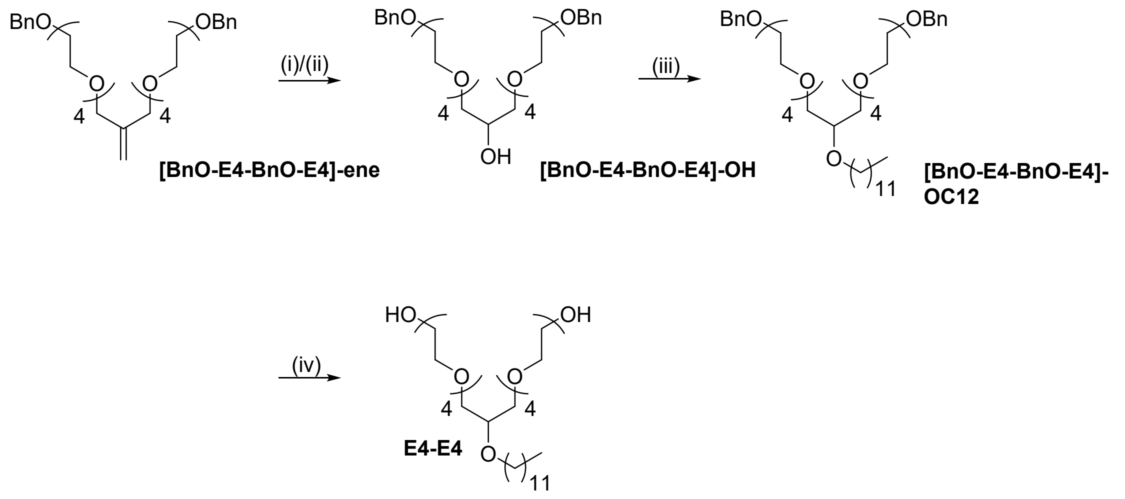

Supplementary Scheme 9. Synthesis of the individual detergent **E4-E4** was achieved in four steps. Reaction conditions were as follows: (i) O_3, DCM/MeOH (*v*:*v*, 1:1), -78 °C, 1h; (ii) $NaBH_4$, -78 °C to RT, 16 h; (iii) 1-bromo-dodecane, NaH (60w%), DMF, 50 °C, 17.5 h; (iv) H_2 (1 bar), PdC (cat.), MeOH/THF (*v*:*v*, 10:1), RT, 24 h. Detailed information about the applied synthesis and purification procedures is given below.

Synthesis of [BnO-E4-BnO-E4]-OH. The starting material **[BnO-E4-BnO-E4]-OH** (3.76 g, 6.05 mmol) was dissolved in dry MeOH (40 mL) and dry DCM (40 mL). The mixture was cooled down to - 78 °C and ozone was passed through it until its color changed to deep blue. Oxygen was then passed through the solution until it became colorless. Sodium borohydride (2.28 g, 60.5 mmol) was added, and the mixture was stirred and allowed to warm up to RT overnight. NH_4Cl (5 mL of a saturated aqueous solution) was added, and the mixture was stirred for 1 h. Organic solvent was removed under reduced pressure and the remaining material was suspended in Brine (100 mL), H_2O (50 mL), and DCM (150 mL). The aqueous layer was extracted with DCM (8 x 200 mL). The combined organic layers were dried over Na_2SO_4, solids were filtered off, and solvent was removed under reduced pressure. Column chromatography (SiO_2, DCM/EtOAc, 4:1 → 2:1 + 10% MeOH) led to the obtainment of **[BnO-E4-BnO-**

E4]-OH (2.38 g, 3.81 mmol, 63%). ^{1}H NMR (500 MHz, $CDCl_3$, ppm): δ = 7.30 – 7.16 (m, 10H), 4.48 (s, 4H), 3.88 (quint, 1H), 3.67 – 3.38 (m, 36H), 3.24 (s, 1H). ^{13}C NMR (126 MHz, $CDCl_3$, ppm): δ = 138.3, 128.3, 127.7, 127.6, 73.2, 72.5, 70.8, 70.6, 70.5, 69.4. MS(ESI+): *m/z* = 647.3398, $C_{33}H_{52}Na_1O_{11}^+$ (calculated = 647.3407).

Synthesis of [BnO-E4-BnO-E4]-OC12. The compound **[BnO-E4-BnO-E4]-OH** (2.38 g, 3.81 mmol) was dissolved in DMF (100 mL) and NaH (60w%, 0.46 g, 11.4 mmol) was added. The mixture was stirred at 50 °C for 90 minutes. 1-Bromododecane (1.83 mL, 7.62 mmol) was added, and the mixture was stirred at 50 °C for 16 h. H_2O (3 mL) was added, and the solvent was removed under reduced pressure. The remaining material was suspended in Brine (100 mL), H_2O (50 mL), and EtOAc (150 mL). The aqueous layer was extracted with EtOAc (8 x 200 mL). The combined organic layers were dried over Na_2SO_4, solids were filtered off, and solvent was removed under reduced pressure. Subsequent column chromatography (SiO_2, DCM/EtOAc, 4:1 → 11:5 + 10% MeOH) gave the desired product **[BnO-E4-BnO-E4]-OC12** (2.03 g, 2.56 mmol, 67%). ^{1}H NMR (500 MHz, $CDCl_3$, ppm): δ = 7.31 – 7.17 (m, 10H), 4.49 (s, 4H), 3.67 – 3.39 (m, 37H), 1.51 – 1.43 (m, 2H), 1.28 – 1.12 (m, 20H), 0.81 (t, 3H). ^{13}C NMR (126 MHz, $CDCl_3$, ppm): δ = 138.4, 128.4, 127.8, 127.7, 77.9, 73.3, 71.4, 71.0, 70.7, 70.6, 69.5, 32.0, 30.2, 29.8, 29.7, 29.6, 29.4, 26.2, 22.8, 14.2. MS(ESI+): *m/z* = 815.5292, $C_{45}H_{76}Na_1O_{11}^+$ (calculated = 815.5285).

Synthesis of E4-E4. The starting material **[BnO-E4-BnO-E4]-OC12** (2.03 g, 2.56 mmol) was dissolved in MeOH (50 mL) and THF (5 mL). Pd/C (Pd on carbon, 10w% loading, 200 mg) was added and the mixture was stirred under hydrogen atmosphere (1 bar) at RT for 48 h. The mixture was passed through a syringe filter (0.45 µm, RC) and the solvent was removed under reduced pressure. Subsequent RP HPLC purification (RPC18, H_2O:MeOH, *v*:*v*, 2:8, flow rate: 20 mL/min) led to the obtainment of the detergent **E4-E4** (1.55 g, 2.53 mmol, 98%). ^{1}H NMR (500 MHz, CD_3OD, ppm): δ = 3.80 – 3.53 (m, 37H), 1.66 – 1.59 (m, 2H), 1.32 – 1.47 (m, 20H), 0.98 (t, 3H). ^{13}C NMR (126 MHz, CD_3OD, ppm): δ = 79.2, 73.7, 72.1, 71.9, 71.6, 71.5, 71.4, 62.2, 33.1, 31.1, 30.8, 30.7, 30.6, 30.5, 27.2, 23.7, 14.5. MS(ESI+): *m/z* = 651.4077, $C_{31}H_{64}K_1O_{11}^+$ (calculated = 651.4086).

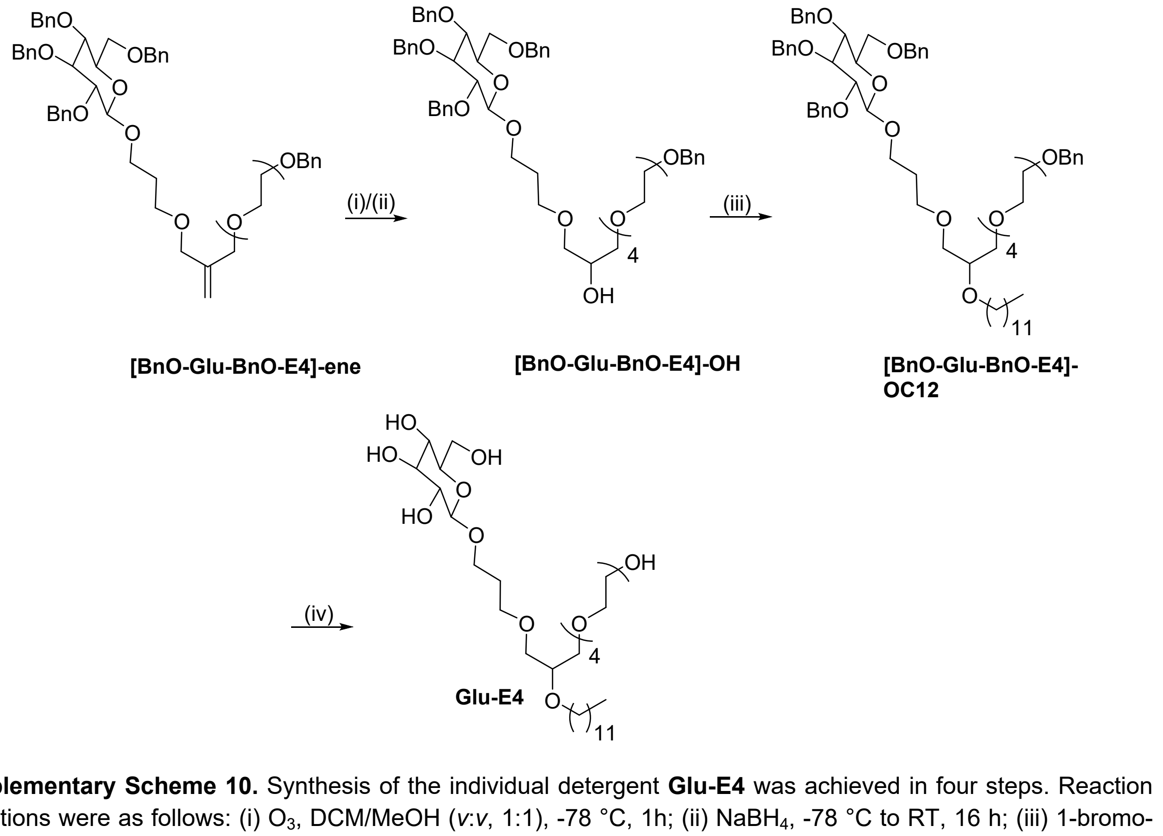

Supplementary Scheme 10. Synthesis of the individual detergent **Glu-E4** was achieved in four steps. Reaction conditions were as follows: (i) O_3, DCM/MeOH (*v*:*v*, 1:1), -78 °C, 1h; (ii) $NaBH_4$, -78 °C to RT, 16 h; (iii) 1-bromo-dodecane, NaH (60w%), DMF, 50 °C, 17.5 h; (iv) H_2 (1 bar), PdC (cat.), MeOH/THF (*v*:*v*, 10:1), RT, 48 h. Detailed information about the applied synthesis and purification procedures is given below.

Synthesis of [BnO-Glu-BnO-E4]-OH. The compound **[BnO-Glu-BnO-E4]-ene** (4.01 g, 4.35 mmol) was dissolved in dry MeOH (40 mL) and dry DCM (40 mL). The mixture was cooled down to -78 °C and ozone was passed through it until its color changed to deep blue. Oxygen was then passed through the solution until it became colorless. Sodium borohydride (1.65 g, 43.5 mmol) was added, and the mixture was stirred and allowed to warm up to RT overnight. NH_4Cl (5 mL of a saturated aqueous solution) was added, and the organic solvent was removed under reduced pressure. The remaining material was suspended in Brine (100 mL), H_2O (50 mL), and DCM (150 mL). The aqueous layer was extracted with DCM (8 x 200 mL). The combined organic layers were dried over Na_2SO_4, solids were filtered off, and solvent was removed under reduced pressure. Column chromatography (SiO_2, DCM/EtOAc, 4:1 → 4:1 + 10 % MeOH) gave the desired product **[BnO-Glu-BnO-E4]-OH** (3.84 g, 4.15 mmol, 95%). ^{1}H NMR (500 MHz, $CDCl_3$, ppm): δ = 7.33 – 7.01 (m, 25H), 4.88 (d, 1H), 4.84 (d, 1H), 4.73 (d, 1H), 4.69 (d, 1H), 4.64 (d, 1H), 4.54 – 4.41 (m, 5H), 4.37 (m, 1H), 3.96 – 3.90 (m, 1H), 3.88 – 3.79 (m, 1H), 3.75 – 3.31 (m, 29H), 2.99 (s, 1H). ^{13}C NMR (126 MHz, $CDCl_3$, ppm): δ = 138.6, 138.5, 138.3, 138.2, 128.4, 128.2, 128.0, 127.9, 127.8, 127.6, 103.8, 84.7, 82.2, 77.9, 75.7, 75.0, 74.9, 74.7, 73.5, 73.2, 72.6, 72.4, 70.7, 70.6, 70.5, 69.5, 69.4, 69.0, 68.9. MS(ESI+): *m/z* = 947.4535, $C_{54}H_{68}Na_1O_{13}^+$ (calculated = 947.4558).

Synthesis of [BnO-Glu-BnO-E4]-OC12. The starting material **[BnO-Glu-BnO-E4]-OH** (3.84 g, 4.15 mmol) was dissolved in DMF (100 mL) and NaH (60w%, 0.50 g, 12.4 mmol) was added. The mixture was stirred at 50 °C for 90 minutes. 1-Bromododecane (2.00 mL, 8.30 mmol) was added dropwise, and the mixture was stirred at 50 °C for another 16 h. H_2O (3 mL) was added, and the solvent was removed under reduced pressure. The solvent was removed under reduced pressure and the remaining material was suspended in Brine (100 mL), H_2O (50 mL), and EtOAc (150 mL). The aqueous layer was extracted with EtOAc (8 x 200 mL) and the combined organic layers were dried over Na_2SO_4. Solids were filtered off and the solvent was removed under reduced pressure. Subsequent column chromatography (SiO_2, DCM/EtOAc, 10:1 → 2:1) led to the obtainment of the desired product **[BnO-Glu-BnO-E4]-OC12** (3.80 g, 3.48 mmol, 84%). ^{1}H NMR (500 MHz, $CDCl_3$, ppm): δ = 7.33 – 7.02 (m, 25H), 4.90 (d, 1H), 4.84 (dd, 1H), 4.73 (d, 1H), 4.69 (dd, 1H), 4.63 (dd, 1H), 4.52 (d, 1H), 4.48 – 4.42 (m, 4H), 4.36 (d, 1H), 3.98 – 3.92 (m, 1H), 3.72 – 3.34 (m, 30H), 1.47 – 1.40 (m, 2H), 1.25 – 1.11 (m, 20H), 0.80 (t, 3H). ^{13}C NMR (126 MHz, $CDCl_3$, ppm): δ = 138.7, 138.6, 138.3, 138.2, 128.4, 128.3, 128.0, 127.9, 127.8, 127.8, 127.6, 103.9, 84.7, 82.1, 77.9, 75.7, 75.0, 74.9, 74.7, 73.5, 73.3, 71.4, 71.3, 70.9, 70.7, 70.6, 69.5, 69.0, 32.0, 30.2, 29.7, 29.6, 29.4, 26.1, 22.7, 14.2. MS(ESI+): *m/z* = 1131.6188, $C_{66}H_{92}K_1O_{13}^+$ (calculated = 1131.6175).

Synthesis of Glu-E4. The compound **[BnO-Glu-BnO-E4]-OC12** (3.80 g, 3.48 mmol) was dissolved in MeOH (50 mL) and THF (5 mL). Pd/C (Pd on carbon, 10w% loading, 600 mg) was added and the mixture was stirred under hydrogen atmosphere at RT for 48 h. The mixture was passed through a syringe filter (0.22 µm, RC) and the solvent was removed under reduced pressure. Subsequent RP HPLC purification (RPC18, H_2O:MeOH, *v*:*v*, 3:7, flow rate: 20 mL/min) led to the obtainment of the desired detergent **Glu-E4** (2.02 g, 3.14 mmol, 90%). ^{1}H NMR (500 MHz, CD_3OD, ppm): δ = 4.32 (d, 1H), 4.05 – 3.99 (m, 1H), 3.88 (dd, 1H), 3.78 – 3.53 (m, 25H), 3.40 – 3.35 (m, 1H), 3.31 – 3.26 (m, 2H), 3.21 (dd, 1H), 1.61 – 1.55 (m, 2H), 1.42 – 1.24 (m, 20H), 0.92 (t, 3H). ^{13}C NMR (126 MHz, CD_3OD, ppm): δ = 104.5, 79.2, 78.0, 75.1, 73.7, 72.0, 71.9, 71.8, 71.6, 71.4, 69.7, 62.8, 62.2, 33.1, 31.1, 30.8, 30.6, 30.5, 27.2, 23.7, 14.5. MS(ESI+): *m/z* = 673.4619, $C_{32}H_{67}N_1O_{13}^+$ (calculated = 673.4607).

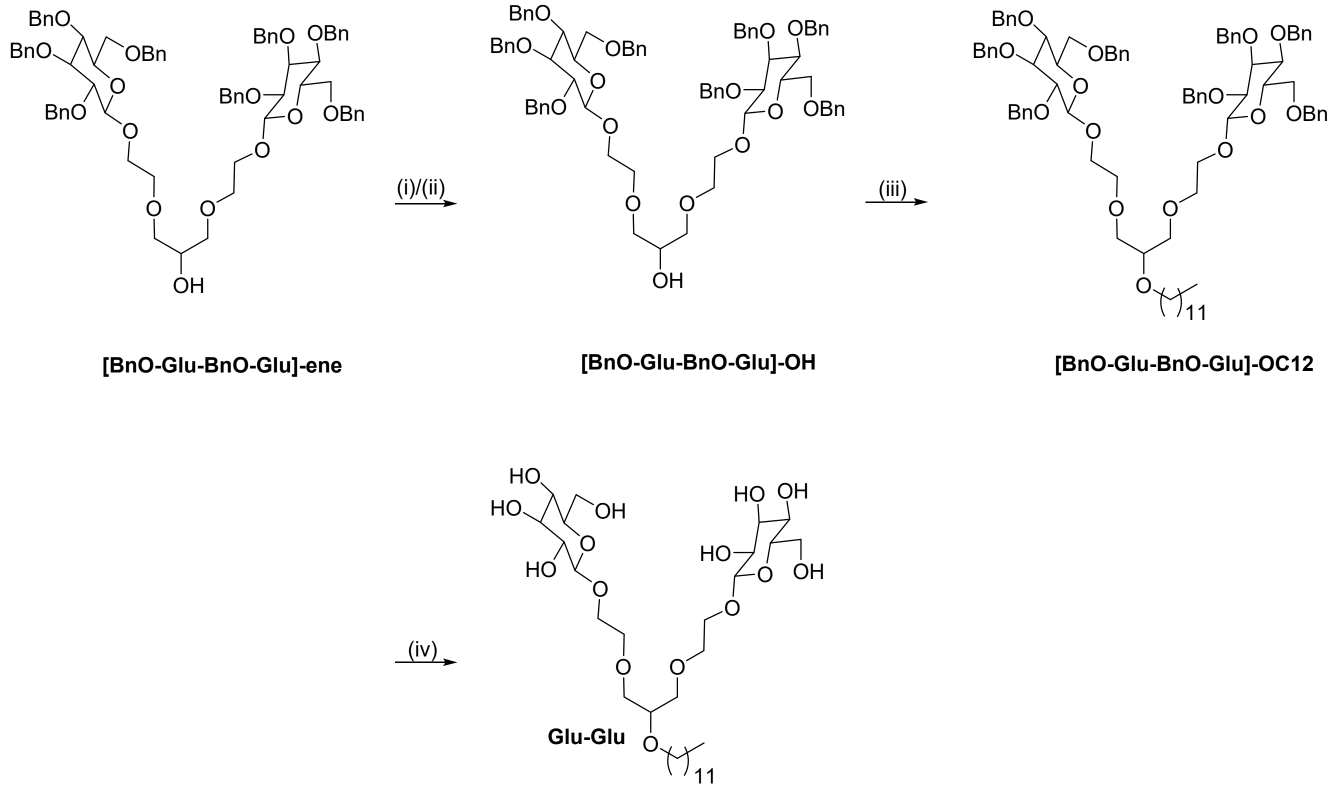

Supplementary Scheme 11. Synthesis of the individual detergent **Glu-Glu** was achieved in four steps. Reaction conditions were as follows: (i) O_3, DCM/MeOH (*v*:*v*, 1:1), -78 °C, 1h; (ii) $NaBH_4$, -78 °C to RT, 16 h; (iii) 1-bromo-dodecane, NaH (60w%), DMF, 50 °C, 17.5 h; (iv) H_2 (5 bar), PdC (cat.), MeOH/THF (*v*:*v*, 10:1), RT, 48 h. Detailed information about the applied synthesis and purification procedures is given below.

Synthesis of [BnO-Glu-BnO-Glu]-OH. The starting material **[BnO-Glu-BnO-Glu]-ene** (2.29 g, 1.88 mmol) was dissolved in dry MeOH (40 mL) and dry DCM (40 mL). The mixture was cooled down to -78 °C and ozone was passed through it until its color changed to deep blue. Oxygen was then passed through the solution until it became colorless. Sodium borohydride (0.71 g, 18.8 mmol) was added, and the mixture was stirred and allowed to warm up to RT overnight. NH_4Cl (5 mL of a saturated aqueous solution) was added, and the organic solvent was removed under reduced pressure. The remaining material was suspended in Brine (100 mL), H_2O (50 mL), and DCM (150 mL). The aqueous layer was extracted with DCM (8 x 200 mL). The combined organic layers were dried over Na_2SO_4, solids were filtered off, and solvent was removed under reduced pressure. Column chromatography (SiO_2, DCM/EtOAc, 1:0 → 4:1) led to the obtainment of the desired product **[BnO-Glu-BnO-Glu]-OH** (1.81 g, 1.48 mmol, 79%). ^1H NMR (500 MHz, $CDCl_3$, ppm): δ = 7.33 – 7.01 (m, 40H), 4.87 (d, 2H), 4.83 (d, 2H), 4.72 (d, 2H), 4.69 (d, 2H), 4.63 (d, 2H), 4.51 (d, 2H), 4.48 – 4.40 (m, 4H), 4.36 (m, 2H), 3.95 – 3.88 (m, 2H), 3.78 (quint, 1H), 3.70– 3.30 (m, 22H), 2.56 (s, 1H). ^{13}C NMR (126 MHz, $CDCl_3$, ppm): δ = 138.8, 138.6, 138.3, 128.5, 128.3, 128.1, 128.0, 127.9, 127.8, 127.7, 103.9, 84.8, 82.2, 78.0, 75.8, 75.1, 75.0, 74.8, 73.6, 72.5, 70.8, 69.5, 69.1, 69.0. MS(ESI+): *m/z* = 1247.5847, $C_{75}H_{84}Na_1O_{15}^+$ (calculated = 1247.5708).

Synthesis of [BnO-Glu-BnO-Glu]-OC12. The compound **[BnO-Glu-BnO-Glu]-OH** (1.81 g, 1.48 mmol) was dissolved in DMF (100 mL) and NaH (60w%, 0.18 g, 4.43 mmol) was added. The mixture was stirred at 50 °C for 90 minutes. 1-Bromododecane (0.71 mL, 2.96 mml) was added, and the mixture was stirred at 50 °C for 16 h. H_2O (3 mL) was added, and the solvent was removed under reduced pressure. The remaining material was suspended in Brine (100 mL), H_2O (50 mL), and EtOAc (150 mL). The aqueous layer was extracted with EtOAc (8 x 200 mL). The combined organic layers were dried over Na_2SO_4, solids were filtered off, and solvent was removed under reduced pressure. Column chromatography (SiO_2, DCM/EtOAc, 1:0 → 10:1) gave the desired product **[BnO-Glu-BnO-Glu]-OC12** (1.85 g, 1.33 mmol, 90%). ^1H NMR (500 MHz, $CDCl_3$, ppm): δ = 7.33 – 7.01 (m, 40H), 4.89 (d, 2H), 4.84 (dd, 2H), 4.73 (d, 2H), 4.69 (dd, 2H), 4.61 (d, 2H), 4.52 (d, 2H), 4.48 – 4.42 (m, 4H), 4.34 (m, 2H), 3.96

– 3.88 (m, 2H), 3.72 – 3.30 (m, 23H), 1.45 – 1.36 (m, 2H), 1.29 – 1.06 (m, 20H), 0.80 (t, 3H). ^{13}C NMR (126 MHz, $CDCl_3$, ppm): δ = 138.7, 138.3, 128.4, 128.3, 128.0, 127.9, 127.7, 103.9, 84.7, 82.2, 78.1, 77.9, 75.8, 75.1, 74.9, 74.7, 73.6, 71.4, 70.7, 69.0, 32.0, 30.2, 29.8, 29.6, 29.5, 26.2, 22.8, 14.2. MS(ESI+): *m/z* = 1415.7632, $C_{87}H_{108}Na_1O_{15}^+$ (calculated = 1415.7586).

Synthesis of Detergent Glu-Glu. The starting material **[BnO-Glu-BnO-Glu]-OC12** (1.85 g, 1.33 mmol) was dissolved in MeOH (50 mL) and THF (5 mL). Pd/C (Pd on carbon, 10w% loading, 300 mg) was added and the mixture was stirred under hydrogen atmosphere (5 bar) at RT for 48 h. The mixture was passed through a syringe filter (0.22 µm, RC) and the solvent was removed under reduced pressure. Subsequent RP HPLC purification (RPC18, H_2O:MeOH, *v*:*v*, 3:7, flow rate: 20 mL/min) led to the obtainment of the desired detergent **Glu-Glu** (0.84 g, 1.25 mmol, 94%). ^1H NMR (500 MHz, CD_3OD, ppm): δ = 4.38 (d, 2H), 4.11 – 4.03 (m, 2H), 3.94 (dd, 2H), 3.84 – 3.58 (m, 13H), 3.46 – 3.32 (m, 6H), 3.27 (t, 2H), 1.63 (m, 2H), 1.48 – 1.30 (m, 20H), 0.97 (t, 3H). ^{13}C NMR (126 MHz, CD_3OD, ppm): δ = 103.2, 77.8, 76.6, 73.8, 70.6, 70.5, 70.3, 70.2, 68.4, 61.5, 31.8, 29.8, 29.5, 29.3, 29.2, 25.9, 22.4, 13.2. MS(ESI+): *m/z* = 695.3825, $C_{31}H_{60}Na_1O_{15}^+$ (calculated = 695.3830).

1.5 Synthesis of detergent mixtures

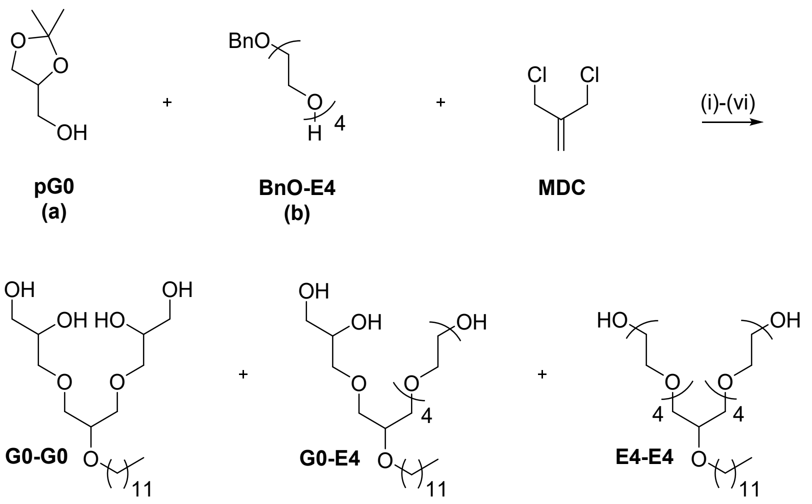

Supplementary Scheme 12. Synthesis of detergent mixture **G0-G0**, **G0-E4**, and **E4-E4** was achieved in six steps (i) – (vi) when starting from **pG0** (**a**), **BnO-E4** (**b**), and **MDC**. Reaction conditions were as follows: (i) NaH (60w%), THF, 50 °C to 80 °C, 23 h; (ii) O_3, DCM/MeOH (*v*:*v*, 1:1), -78 °C, 1h; (iii) $NaBH_4$, -78 °C to RT, 16 h; (iv) NaH (60w%), 1-bromododecane, DMF, 50 °C to RT, 17 h; (v) H_2 (5 bar), Pd/C (cat.), MeOH, RT, 19 h; (vi) HCl (37w%), MeOH, RT, 23 h. Detailed information about the synthesis and purification procedures is given below.

Synthesizing a Mixture of Detergents G0-G0, G0-E4, and E4-E4. The starting materials **pG0** (0.45 g, 3.40 mmol) and **BnO-E4** (0.96 g, 3.40 mmol) were dissolved in dry THF (60 mL). NaH (60w%, 0.42 g, 10.4 mmol) and catalytic amounts of 15-crown-5 were added. The mixture was stirred at 50 °C for 1 h. **MDC** (0.39 mL, 3.40 mmol), catalytic amounts of 18-crown-6, and catalytic amounts of potassium iodide were added and the mixture was stirred at 80 °C for 23 h. The mixture was allowed to cool down to RT and H_2O (1 mL) was added. The solvent was removed under reduced pressure. The remaining material was suspended in Brine (270 mL), H_2O (30 mL), and DCM (300 mL). The aqueous layer was extracted with DCM (6 x 50 mL). The organic layers were dried over Na_2SO_4, solids were filtered off, and the solvent was removed under reduced pressure. The so-obtained raw product (1.92 g) was dissolved in dry MeOH (40 mL) and dry DCM (40 mL). The mixture was cooled down to - 78 °C and ozone was passed through it until its color changed to deep blue. Oxygen was then passed through the solution until it became colorless. Sodium borohydride (1.27 g, 33.6 mmol) was added and the mixture was stirred and allowed to warm up to RT overnight. NH_4Cl (2 mL of a saturated aqueous solution) was added and the mixture was stirred for another five minutes. Solvent was removed under reduced pressure and the remaining material was suspended in Brine (25 mL), H_2O (3 mL), and DCM (30 mL).

The aqueous layer was extracted with DCM (6 x 15 mL). The organic layers were dried over Na_2SO_4, solids were filtered off, and the solvent was removed under reduced pressure. The so-obtained raw product (1.91 g) was dissolved in DMF (25 mL). NaH (60w%, 0.40 g, 10.0 mmol) was added and the mixture was stirred at 50 °C for 1 h. 1-Bromododecane (1.61 mL, 6.70 mmol) was added and the mixture was stirred at RT for 16 h. H_2O (2 mL) was added and the solvent was removed under reduced pressure. The remaining material was suspended in Brine (25 mL), H_2O (3 mL), and DCM (30 mL). The aqueous layer was extracted with DCM (6 x 15 mL). The organic layers were dried over Na_2SO_4, solids were filtered off, and the solvent was removed under reduced pressure. The raw product was filtrated over a short silica gel column (SiO_2, DCM/EtOAc + MeOH, 1:0 → 4:1 + 10%) to remove excess of mineral oil and 1-bromododecane. The so-obtained raw product (1.96 g) was dissolved in MeOH (8 mL). Pd/C (Pd on carbon, loading 10w%, 200 mg) was added, and the mixture was stirred under hydrogen atmosphere (5 bar) at RT for 19 h. The mixture was passed through a syringe filter (0.45 µm, RC) and the solvent was removed under reduced pressure. The so-obtained raw product (1.59 g) was dissolved in MeOH (80 mL) and HCl (100 µL of a 37w% aqueous solution) was added. The mixture was stirred at RT for 23 h and NEt_3 (100 µL) was added. The solvent was removed under reduced pressure to obtain a mixture of the detergents **G0-G0**, **G0-E4**, and **E4-E4** (1.87 g). Fingerprint NMR Data (obtained from product mixture): ^{1}H NMR (700 MHz, CD_3OD, ppm): δ = 3.84 – 3.80 (m, 0.35H), 3.74 – 3.69 (m, 3.09H), 3.68 – 3.52 (m, 3.83H), 3.31 – 3.27 (m, 2.44H), 1.65 – 1.60 (m, 0.68H), 1.41 – 1.36 (m, 8.49H), 0.99 – 0.96 (m, 1H).[3†] ^{13}C NMR (176 MHz, CD_3OD, ppm): δ = 73.9, 73.9, 73.8, 72.2, 72.1, 72.0, 71.8, 71.5, 71.4, 71.3, 47.8, 33.0, 33.0, 31.1, 30.7, 30.7, 30.5, 30.4, 27.18, 23.7, 14.5, 14.4, 9.2. MS(ESI+, obtained from product mixture): **G0-G0**, *m/z* = 431.2975, $C_{21}H_{44}Na_1O_7^+$ (calculated = 431.2979); **G0-E4**, *m/z* = 533.3667, $C_{26}H_{54}Na_1O_9^+$ (calculated = 533.3660); **E4-E4**, *m/z* = 635.4340, $C_{31}H_{64}Na_1O_{11}^+$ (calculated = 635.4341).

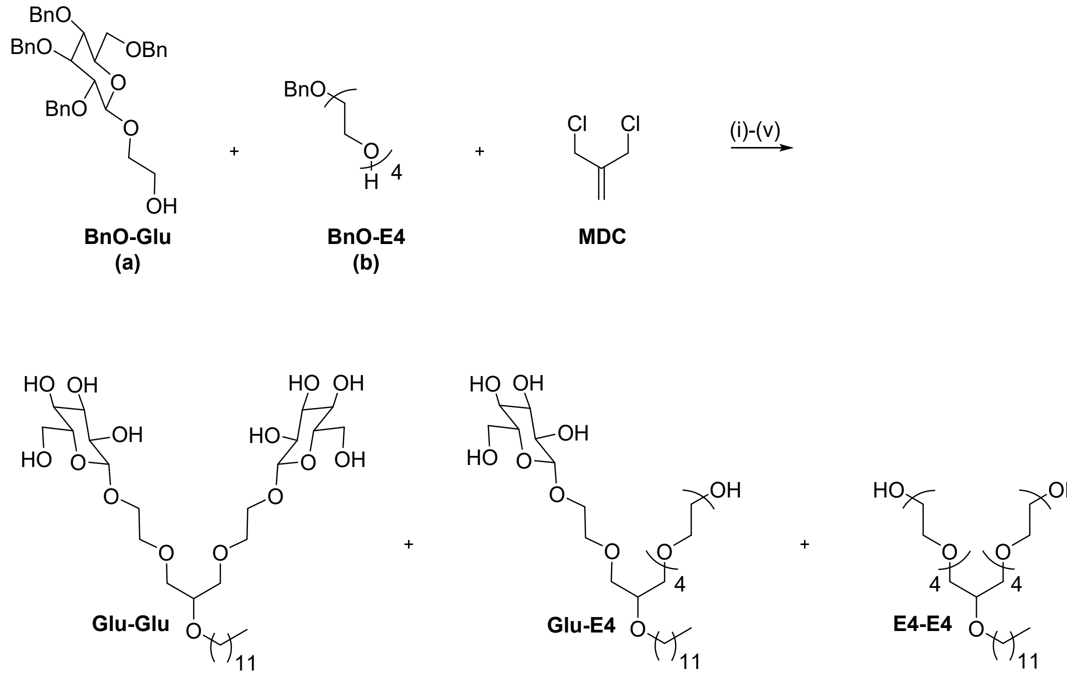

Supplementary Scheme 13. Synthesis of detergent mixture **E4-E4**, **Glu-E4**, and **Glu-Glu** was achieved in five steps (i) – (v) when starting from **BnO-Glu** (**a**), **BnO-E4** (**b**), and **MDC**. Reaction conditions were as follows: (i) NaH (60w%), THF, 50 °C to 80 °C, 19 h; (ii) O_3, DCM/MeOH (*v*:*v*, 1:1), -78 °C, 1h; (iii) $NaBH_4$, -78 °C to RT, 16 h; (iv) NaH (60w%), 1-bromododecane, DMF, 50 °C to RT, 17 h; (v) H_2 (5 bar), Pd/C (cat.), MeOH, RT, 74 h. Detailed information about the applied synthesis and purification procedures is given below.

Synthesizing a Mixture of Detergents 4, 5, and 6. The starting materials **BnO-Glu** (1.79 g, 3.10 mmol) and **BnO-E4** (0.87 g, 3.10 mmol) were dissolved in dry THF (60 mL). NaH (60w%, 0.37 g, 9.20 mmol) and catalytic amounts of 15-crown-5 were added. The mixture was stirred at 50 °C for 1 h. **MDC**

[3†] For the NMR fingerprint analysis, this ^{1}H NMR integral was set to 1.

(0.35 mL, 3.00 mmol), catalytic amounts of 18-crown-6, and catalytic amounts of potassium iodide were added. The mixture was stirred at 80 °C for 19 h. The mixture was allowed to cool down to RT and H_2O (2 mL) was added. The solvent was removed under reduced pressure and the remaining material was suspended in Brine (100 mL), H_2O (100 mL), and DCM (200 mL). The aqueous layer was extracted with DCM (5 x 50 mL). The combined organic layers were dried over Na_2SO_4, solids were filtered off, and solvent was removed under reduced pressure. Excess of mineral oil were removed by column chromatography (SiO_2, DCM/EtOAc, 12:1 → 4:1 + 10% MeOH). The so-obtained raw product (2.76 g) was dissolved in dry MeOH (35 mL) and dry DCM (35 mL). The mixture was cooled down to - 78 °C and ozone was passed through it until its color changed to deep blue. Oxygen was then passed through the solution until it became colorless. Sodium borohydride (1.15 g, 30.4 mmol) was added, and the mixture was stirred and allowed to warm up to RT overnight. NH_4Cl (2 mL of a saturated aqueous solution) was added, and the mixture was stirred for ten minutes. Solvent was removed under reduced pressure and the remaining material was suspended in Brine (47 mL), H_2O (3 mL), and DCM (50 mL). The aqueous layer was extracted with DCM (6 x 30 mL). The combined organic layers were dried over Na_2SO_4, solids were filtered off, and solvent was removed under reduced pressure. The so-obtained raw product (2.61 g) was dissolved in DMF (25 mL). NaH (60w%, 0.36 g, 9.20 mmol) was added and the mixture was stirred at 50 °C for 1 h. 1-Bromododecane (1.46 mL, 6.10 mmol) was added, and the mixture was stirred at RT for 16 h. H_2O (2 mL) was added, and the solvent was removed under reduced pressure. The remaining material was suspended in Brine (44 mL), H_2O (6 mL), and EtOAc (50 mL). The aqueous layer was extracted with EtOAc (6 x 15 mL) and the combined organic layers were dried over Na_2SO_4. Solids were filtered off and the solvent was removed under reduced pressure. Excess of 1-bromododecane was removed by a filtration over silica gel (SiO_2, DCM/EtOAc, 1:0 → 4:1 + 15% MeOH). The so-obtained raw product (2.68 g) was dissolved in MeOH (4.5 mL) and THF (4.5 mL). Pd/C (Pd on carbon, 10w% loading, 300 mg) was added and the mixture was stirred under hydrogen atmosphere (5 bar) at RT for 74 h. The mixture was passed through a syringe filter (0.45 µm, RC). The solvent was removed under reduced pressure in order to obtain a mixture of detergents **E4-E4**, **Glu-E4**, and **Glu-Glu** (1.63 g). Fingerprint NMR Data (obtained from product mixture): ^{1}H NMR (700 MHz, CD_3OD, ppm): δ = 4.31 – 4.37 (m, 0.22H), 4.11 – 4.06 (m, 0.22H), 3.97 – 3.93 (m, 0.23H), 3.85 – 3.59 (m, 7.45H), 3.47 – 3.43 (m, 0.58H), 3.40 – 3.35 (m, 0.52H), 3.31 – 3.27 (m, 0.23H), 1.68 – 1.60 (m, 0.67H), 1.50 – 1.34 (m, 6.33H), 1.01 – 0.97 (m, 1H).[4†] ^{13}C NMR (176 MHz, CD_3OD, ppm): δ = 104.3, 77.8, 74.9, 73.6, 71.8, 71.5, 71.4, 71.3, 62.1, 49.8, 33.0, 31.0, 30.7, 30.5, 30.4, 27.1, 23.6, 14.4. MS(ESI+, obtained from product mixture): **E4-E4**, *m/z* = 635.4375, $C_{31}H_{64}Na_1O_{11}^+$ (calculated = 635.4341); **Glu-E4**, *m/z* = 665.4119, $C_{31}H_{62}Na_1O_{13}^+$ (calculated = 665.4083); **Glu-Glu**, *m/z* = 695.3872, $C_{31}H_{60}Na_1O_{15}^+$ (calculated = 695.3824).

[4†] For the NMR fingerprint analysis, this ^{1}H NMR integral was set to 1.

1.6 Hydrophilic-lipophilic balance (HLB) calculation

The hydrophilic-lipophilic balance (HLB) of our detergents was calculated using the equation (1) in which individual parameters are defined as follows: *molecular weight of the tail* (MW_{tail}) and *molecular weight of the detergent* (MW). HLB values are summarized in Table S2 (see Supplementary Tables).

$$HLB = 20 \cdot \left(1 - \frac{MW_{tail}}{MW}\right) \tag{1}$$

1.7 Packing parameter calculation

The packing parameter was calculated using equation (2) in which individual parameters are defined as follows: *volume of the tail* (V), *length of the tail* (L), *head group area* (A), and *packing parameter* (p).[2]

$$p = \frac{V}{L \cdot A} \tag{2}$$

In the packing parameter calculation, the *head group area* (A) is often defined as the equilibrium area of the detergent head group occupied at the surface of a detergent aggregate, for example, a micelle. The equilibrium head group area can be determined by means of surface tension measurements.[3] Here, we apply simple geometric calculations to approximate the molecular shape of our detergents based on their molecular structure. To do so, we modeled the detergent head groups (without tail) using the MM2 force field as implemented in ChemBio3D v14.0 (PerkinElmer) and determined their rotationally averaged collision cross section (CCS) using a projection approximation algorithm.[4] From equation (2) follows that, given a constant hydrophobic tail, only the *head group area* (A) determines the *packing parameter* or "molecular shape" of a detergent. In this view if, one wants to compare relative changes in *packing parameter* or "molecular shape," we assume, that one can use either the equilibrium head group area in solution or the CCS of the detergent head group determined in vacuum. The volume of the hydrophobic detergent tails were approximated by using the van der Waals volume calculation method of Zhao *et al*.[5] The tail length was estimated with ChemBio3D v14.0 (PerkinElmer). Packing parameters based on CCS calculations are summarized in Table S3 (see Supplementary Tables). Errors of CCS data are below one percent.

1.8 Critical aggregation concentration

Critical aggregation concentration values were determined by dynamic light scattering using a previously established protocol:[1, 6] Dilution series with detergent concentrations between 10^{-8} and 10^{-2} mol·L^{-1} were prepared in ultrapure water, which was obtained from a Milli-Q system (18.2 MΩ·cm). All samples were filtered (0.22 µm, RC) and equilibrated for at least for 16 hours at room temperature (approximately 22 °C) prior analysis. The samples were analysed in cuvettes (Quartz Suprasil, width x length: 2 mm x 10 mm) using a Zetasizer Nano-ZS ZEN3600 (Malvern, UK). The instrumental parameters were as follows: material (polystyrene latex), dispersant (water), sample viscosity parameters (use dispersant viscosity as sample viscosity), temperature (22.5 °C), equilibration time (120 s), cell type (quartz cuvettes), measurement angle (173° backscatter), measurement duration (manual), number of runs (11), run duration (10 s), number of measurements (3), delay between the measurements (0 s), data processing (general purpose, normal resolution). The derived count rate values obtained from three measurements per concentration were averaged. The unit of the derived count rate is kilo counts per second (kcps). The logarithm of the derived count rate was plotted against the logarithm of the concentration. The double logarithmic plots showed two characteristic regions: 1.) a flat region with low count rates at lower detergent concentrations and 2.) a linear growth of the count rate at higher

concentrations. Both regions were fitted to linear functions and the intersection was taken as the *cac* value. The *cac* data are summarized in Table S4.

1.9 Circular dichroism spectroscopy

The secondary structure of AmtB, AqpZ, and OmpT in different detergents was investigated by circular dichroisms (CD) spectroscopy following a previously established procedure:[1, 6a, 7] Briefly, proteins were purified with reference detergents, e.g., *n*-dodecyl-ß-D-maltoside (AqpZ, AmtB) or lauryldimethylamine-N-oxide (OmpT). The detergent environment of the proteins was exchanged for scalable hybrid detergents over desalting columns (for details see Ref. [1]). Protein solutions were adjusted to similar concentrations and analysed by CD spectroscopy using the following parameters: Protein solutions were loaded into cuvettes (Quartz Suprasil, volume = 300 µL, layer thickness = 1 mm). The CD spectrometer (Chirascan, USA) was purged with nitrogen overnight and turned on 30 min before use together with the sample cooler. The following experimental parameters were used: temperature (22.5 °C), wavelength range (200–260 nm), step size (0.5–1 nm), scan speed (0.5 s/point), bandwidth (1 nm), and repeats per sample (4). The average CD intensity of four scans was plotted against the wavelength. Detergent-containing CD spectroscopy buffers were used as blanks. Data were acquired with Pro-Data Chirascan 4.5 and analysed with Origin V9.1.

2. Supplementary figures

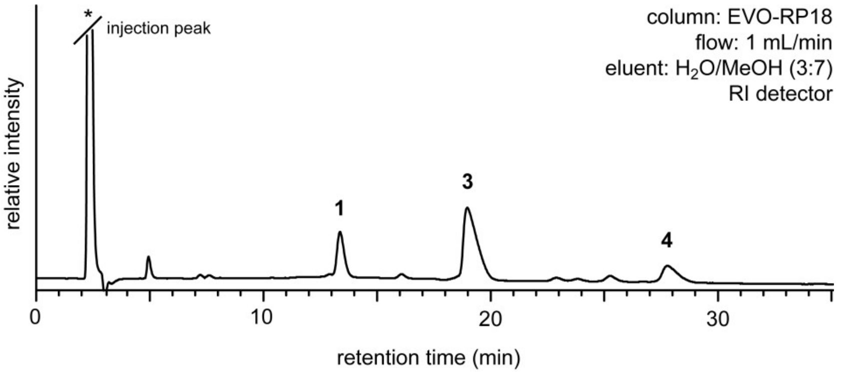

Figure S1. Chromatogram obtained from analytical, reversed phase HPLC analysis of a trimeric detergent mixture that was synthesized in one go. The mixture includes three detergents, e.g. **G0-G0** (**1**), **G0-E4** (**3**), **E4-E4** (**4**). Injection peak is labelled with an asterisk.

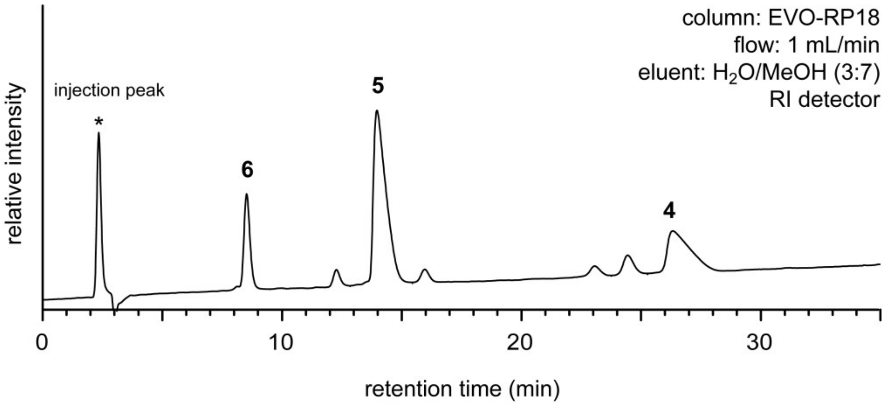

Figure S2. Chromatogram obtained from analytical, reversed phase HPLC analysis of a trimeric detergent mixture that was synthesized in one go. The mixture includes three detergents, e.g. **Glu-Glu** (**6**), **Glu-E4** (**5**), **E4-E4** (**4**). Injection peak is labelled with an asterisk.

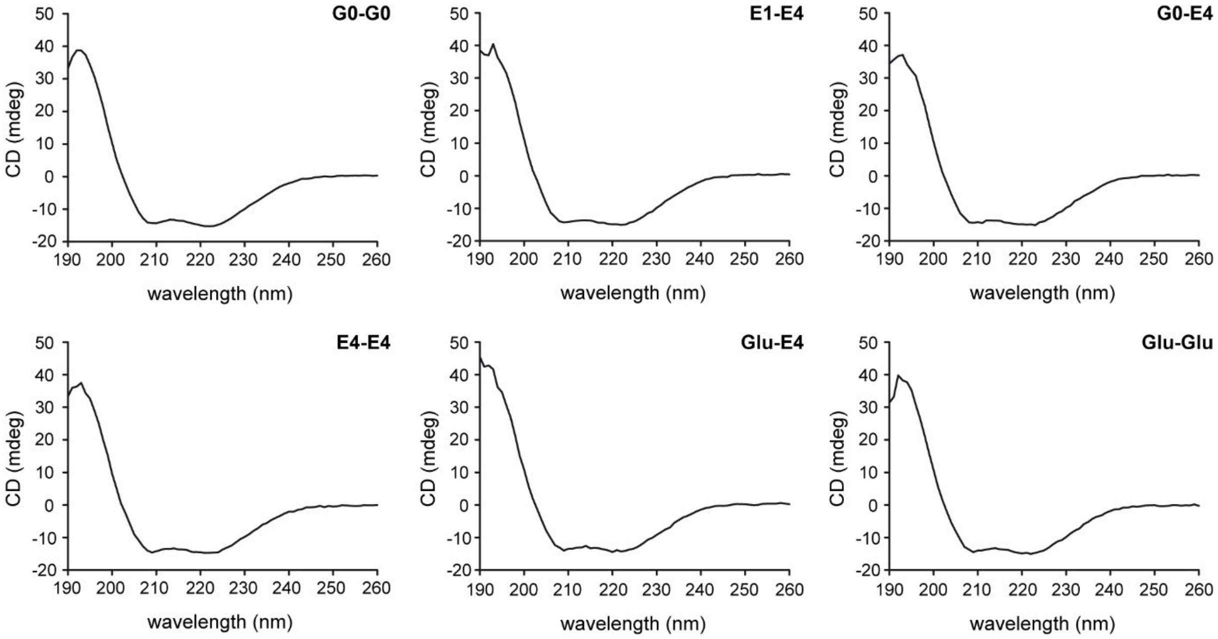

Figure S3. CD spectra of AmtB (2.12 μM) upon detergent exchange from DDM into scalable hybrid detergents. Similar overall fold was preserved in all cases, suggesting that scalable hybrid detergents can solubilize and stabilize the native fold of membrane proteins.

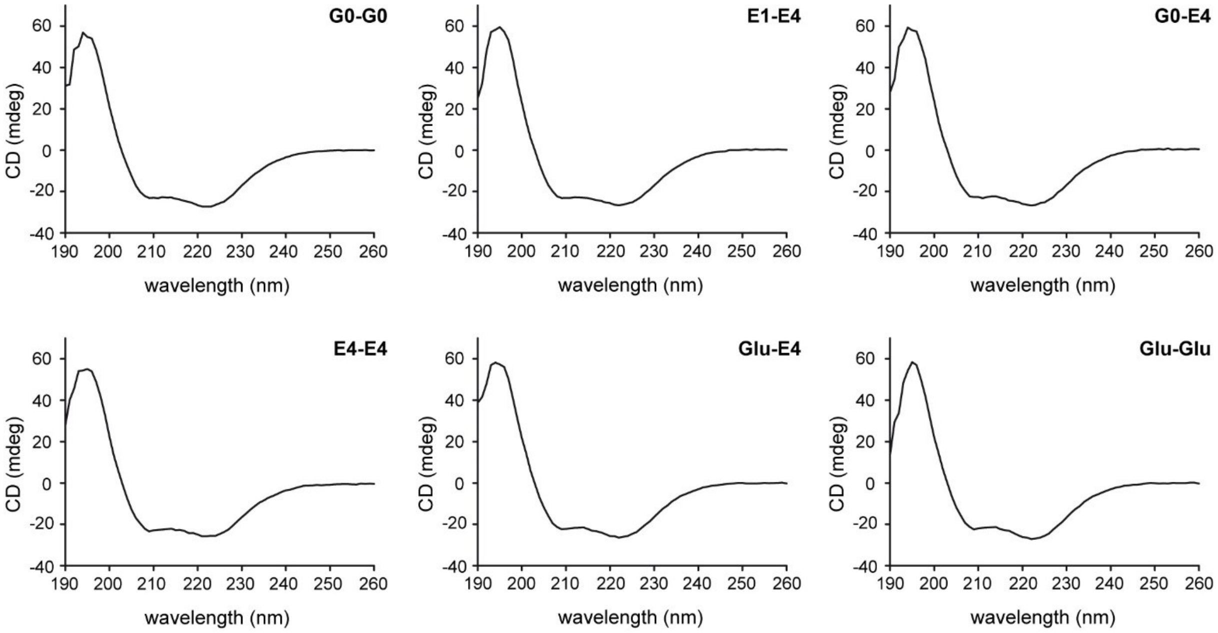

Figure S4. CD spectra of *Rs*TSPO (7.95 μM) upon detergent exchange from DDM into scalable hybrid detergents. Similar overall fold was preserved in all cases, suggesting that scalable hybrid detergents can solubilize and stabilize the native fold of membrane proteins.

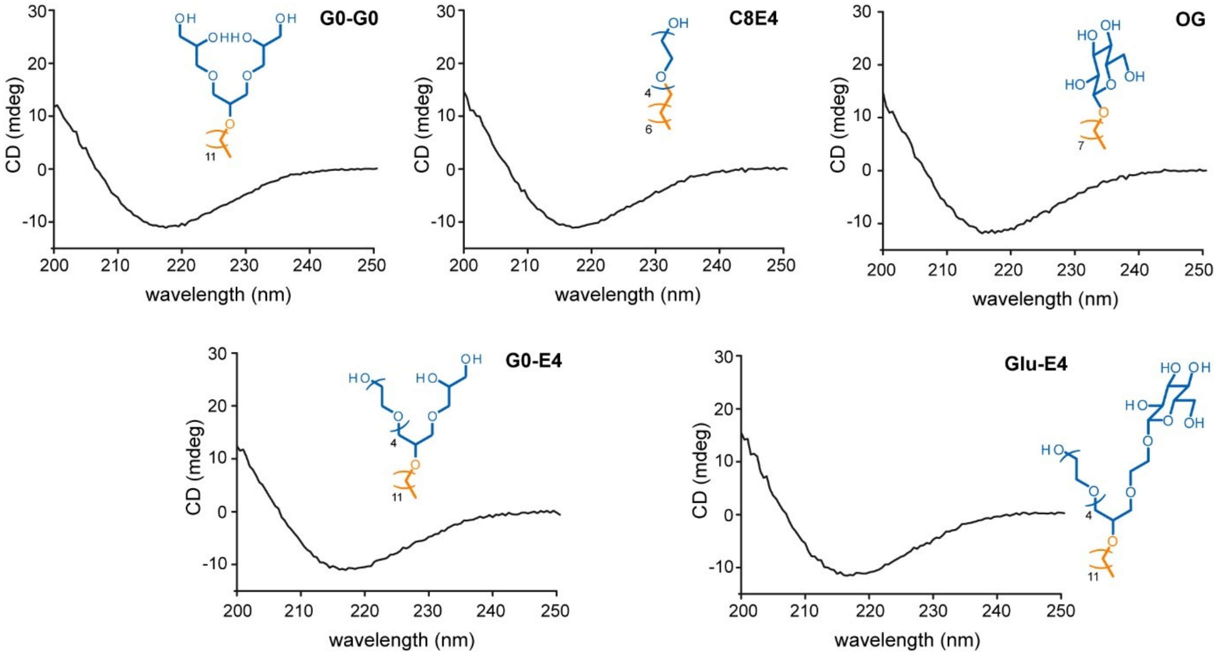

Figure S5. CD spectra of OmpT (4.77 μM) upon detergent exchange from LDAO into scalable hybrid detergents. Similar overall fold was preserved in all cases, suggesting that scalable hybrid detergents can solubilize and stabilize the native fold of membrane proteins.

3. Supplementary tables

Table S1. Summary of product ratios obtained from different combinatorial head group syntheses. Product ratios were determined from the raw products after aqueous work-up by means of ^{13}C NMR (inverse-gated mode).

building block combinations	relative product intensities (aa:ab:bb)
**pG0 + BnO-E4**	1:3:1
**BnO-Glu + BnO-E4**	1:3:1
**BnO-E1+ BnO-E4**	1:3:1

detergent	molecular weight (g/mol)	molecular weight of the tail (g/mol)	HLB
[G1] OGD (**G0-G0**)	408.5	169.3	11.7
**E4-E1**	480.6	169.3	12.9
**E4-G0**	510.7	169.3	13.3
**E4-E4**	612.8	169.3	14.4
**E4-Glu**	642.8	169.3	14.7
**Glu-Glu**	672.8	169.3	14.9
G2 OGD	704.5	169.3	15.2

Table S2. Summary of data used for the calculation of hydrophilic-lipophilic balance (HLB). Detergents are listed in order of increasing molecular weight (**G0-G0** → [G2] OGD).

Table S3. Summary of data used for the calculation of packing parameters, including the CCS of the head groups, volumes of the tails, length of the tails, and packing parameters. Detergents are listed in order of increasing molecular weight (**G0-G0** → [G2] OGD).

Detergent	CCS of the head group (nm^2)	volume of the tail (nm^3)	length of the tail (nm)	packing parameter
[G1] OGD (**G0-G0**)	1.023	0.215	1.6	0.13
**E4-E1**	1.118	0.215	1.6	0.12
**E4-G0**	1.266	0.215	1.6	0.10
**E4-E4**	1.536	0.215	1.6	0.09
**E4-Glu**	1.442	0.215	1.6	0.09
**Glu-Glu**	1.455	0.215	1.6	0.09
G2 OGD	1.770	0.215	1.6	0.08

Table S4. Summary of critical aggregation concentration (cac) values of detergents. The cac values were determined by dynamic light scattering or were obtained from the commercial supplier.[a]

Detergent	cac (mM)	typical concentration in purification or assay buffers (2x cac)
[G1] OGD (**G0-G0**)	0.5	1
**E4-E1**	1	2
**E4-G0**	0.4	0.8
**E4-E4**	0.5	1
**E4-Glu**	0.5	1
**Glu-Glu**	0.5	1
**OG**	23[a]	46
**C8E4**	8[a]	16

[a] obtained from commercial supplier

4. Literature

[1] L. H. Urner, I. Liko, H.-Y. Yen, K. K. Hoi, J. R. Bolla, J. Gault, F. G. Almeida, M.-P. Schweder, D. Shutin, S. Ehrmann, R. Haag, C. V. Robinson, K. Pagel, *Nat. Commun.* **2020**, *11*, 564.

[2] J. N. Israelachvili, D. J. Mitchell, B. W. Ninham, *J. Chem. Soc., Faraday Trans. 2* **1976**, *72*, 1525-1568.

[3] P. F. Garrido, P. Brocos, A. Amigo, L. García-Río, J. Gracia-Fadrique, Á. Piñeiro, *Langmuir* **2016**, *32*, 3917-3925.

[4] G. von Helden, M. T. Hsu, N. Gotts, M. T. Bowers, *J. Phys. Chem.* **1993**, *97*, 8182-8192.

[5] Y. H. Zhao, M. H. Abraham, A. M. Zissimos, *J. Org. Chem.* **2003**, *68*, 7368-7373.

[6] aL. H. Urner, M. Schulze, Y. B. Maier, W. Hoffmann, S. Warnke, I. Liko, K. Folmert, C. Manz, C. V. Robinson, R. Haag, K. Pagel, *Chem. Sci.* **2020**, *11*, 3538-3546; bL. H. Urner, I. Liko, K. Pagel, R. Haag, C. V. Robinson, *BBA - Biomembranes* **2022**, *1864*, 183958.

[7] L. H. Urner, E. Mohammadifar, K. Ludwig, D. Shutin, F. Fiorentino, I. Liko, F. G. Almeida, D. Kutifa, R. Haag, C. V. Robinson, *ACS Appl. Polym. Mater.* **2021**, *3*, 5903-5911.
